# Direct and Sustainable Ammonia Synthesis from Air and Water with Sulfur-Deficient MoS_2_ Piezocatalysts

**DOI:** 10.1021/acsnano.5c11903

**Published:** 2025-10-06

**Authors:** Yu-Ching Chen, Yin-Song Liao, Po-Han Chen, Jyh-Pin Chou, Cheng-Kuo Tsai, Yi-Dong Lin, Yan-Gu Lin, Yu-Ren Peng, Jyh Ming Wu

**Affiliations:** 1 Department of Materials Science and Engineering, 34881 National Tsing Hua University, 101, Section 2 Kuang Fu Road, Hsinchu 300, Taiwan; 2 Ph.D. Program in Prospective Functional Materials Industry, 34881 National Tsing Hua University, 101, Section 2 Kuang Fu Road, Hsinchu 300, Taiwan; 3 High Entropy Materials Center, 34881 National Tsing Hua University, 101, Section 2 Kuang Fu Road, Hsinchu 300, Taiwan; 4 Tsing Hua Interdisciplinary Program, 34881 National Tsing Hua University, 101, Section 2 Kuang Fu Road, Hsinchu 300, Taiwan; 5 Graduate School of Advanced Technology, 33561 National Taiwan University, Taipei 106319, Taiwan; 6 Department of Physics, National Changhua University of Education, No. 1, Jin-De Road, Changhua 500, Taiwan; 7 Emergency Response Information Center, 34883 National Yunlin University of Science and Technology, Douliu City, Yunlin County 64002, Taiwan; 8 Institute of Pioneer Semiconductor Innovation, National Yang Ming Chiao Tung University, Hsinchu 300, Taiwan; 9 57815 National Synchrotron Radiation Research Center, 101 Hsin-Ann Road, Hsinchu Science Park, Hsinchu 300, Taiwan

**Keywords:** MoS_2_, sulfur vacancies, piezocatalysis, atmospheric nitrogen reduction reaction, sustainable energy

## Abstract

Eco-friendly ammonia (NH_3_) production is critical for advancing sustainable agriculture and industry. This study introduces a sustainable, cleaner approach using MoS_2_ nanoflowers (NFs) to synthesize NH_3_ directly from water and air without the need for sacrificial agents. The advanced design leverages double sulfur vacancies (V2s) in MoS_2_ NFs (V2s-MoS_2_ NFs) and their piezoelectric properties, achieving a noteworthy production efficiency of 8374.8 ± 140.1 μmol L^–1^ g^–1^ h^–1^ (absolute production rate of 0.84 ± 0.01 μmol h^–1^). This outperforms most existing photocatalysts and piezocatalysts and rivals advanced electrocatalysts. The catalyst demonstrated exceptional stability, producing 36.55 mmol L^–1^ g^–1^ (equivalent to an absolute yield of 3.655 μmol) with N_2_ and 26.03 mmol L^–1^ g^–1^ (equivalent to an absolute yield of 2.603 μmol) with air over 8 h. In situ Raman spectroscopy revealed intensifying peaks at ∼819 and 993 cm^–1^ under N_2_ gas, attributed to Mo–N stretching vibrations. Additionally, in situ diffuse reflectance infrared Fourier-transform spectroscopy showed N_2_ adsorption configurations, including side-on adsorption, indicative of N≡N bond elongation on the catalyst surface. Density functional theory calculations corroborated these findings, illustrating how unpaired Mo d orbital electrons near sulfur vacancies activate N_2_ dissociation via backdonation to N_2_’s antibonding π orbitals. This research highlights the transformative potential of piezocatalytic systems for nitrogen reduction reactions using atmospheric N_2_ and water, providing a basis for sustainable energy solutions.

## Graphical abstract

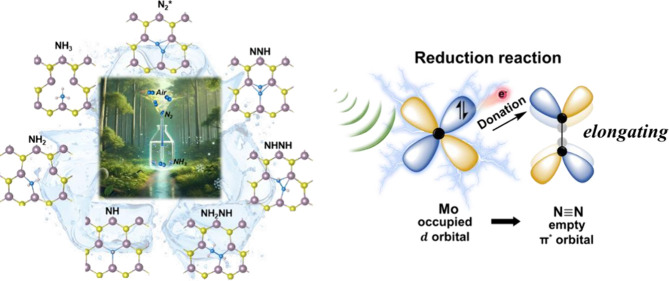

## 1 Introduction

Ammonia (NH_3_) is an essential compound with wide-ranging applications, from agriculture to energy storage, and serves as a promising carbon-free fuel for the future.^1,2^ The century-old Haber-Bosch process, which revolutionized NH_3_ production, relies on high temperatures and pressures, consuming vast amounts of energy and emitting significant greenhouse gases. As global energy demands increase, there is an urgent need for greener, more sustainable methods to produce NH_3_ under ambient conditions. Various strategies have been explored to enhance N_2_-to-NH_3_ conversion by electrosynthesis^3,4^ and photocatalytic N_2_ fixation.^5,6^ However, the direct utilization of atmospheric nitrogen (N_2_) for NH_3_ production has been an enduring scientific challenge, primarily due to the extremely strong N≡N bond (940 kJ mol^–1^) that makes N_2_ molecules highly stable and resistant to conversion.

To tackle these challenges, we introduce a groundbreaking method: converting atmospheric N_2_ directly from air into NH_3_ using only water and a novel MoS_2_-based piezocatalyst under ambient conditions. Our process not only efficiently utilizes N_2_ but also uniquely harnesses air, offering a simple and environmentally friendly alternative to conventional methods. This eliminates the need for high-purity N_2_ gas and sacrificial agents typically required for precise electrocatalyst systems.^1,7^ Despite the inherent difficulty of activating N_2_ due to its chemical inertness, our work demonstrates that defect engineering, specifically the introduction of sulfur vacancies, can drive the N_2_ reduction process through the synergistic effect of piezoelectricity. By engineering double sulfur vacancies (V2s) in MoS_2_ nanoflowers (NFs), we developed a catalyst that leverages its intrinsic piezoelectric properties to significantly enhance the nitrogen reduction reaction (NRR). When subjected to mechanical strain, these MoS_2_ NFs generate a strong piezoelectric field that efficiently separates electron–hole pairs, facilitating the conversion of N_2_ to NH_3_ with unprecedented efficiency—all under ambient conditions without the need for high temperatures, pressures, or sacrificial agents.

As illustrated in Figure 1a, air-to-ammonia conversion demonstrates how air can serve as the sole reagent in water during the NRR facilitated by piezocatalytic MoS_2_ NFs. In Figure 1b, our density functional theory (DFT) simulations show that applying strain—from compression to tension—on the V2s-MoS_2_ surface alters the orientation of adsorbed N_2_ molecules, favoring a side-on configuration that enhances electron backdonation from unpaired Mo 3d orbitals to the antibonding π* orbitals of N_2_. This interaction weakens the N≡N bond,^8,9^ elongating its length, and lowering the energy barrier for dissociation under mild conditions (Figure 1c).^5,8^ As shown in Figure 1d, our work achieves an exceptional yield of 8374.8 ± 140.1 μmol L^–1^ g^–1^ h^–1^ (absolute production rate of 0.84 ± 0.01 μmol h^–1^), positioning it among the most state-of-the-art techniques. It surpasses most reported photocatalytic and piezo-coupled catalytic performances and is the first to operate solely under the piezoelectric effect, without a sacrificial agent. (Supporting Information, Table S1).^10–22^

**Figure 1:**
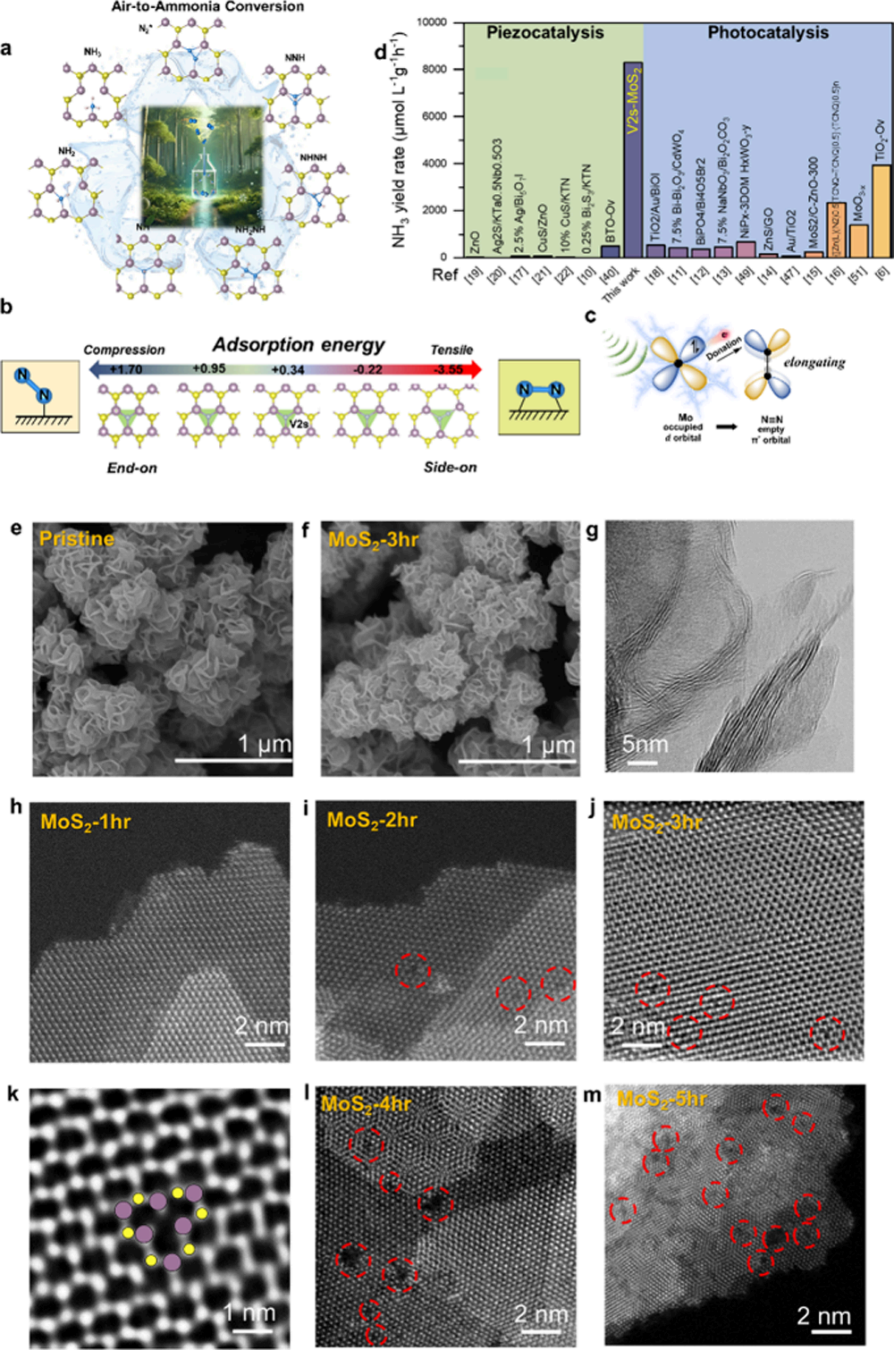
Role of sulfur vacancies in catalyzing N_2_ hydrogenation to NH_3_. (a) Schematic illustration of the V2s-MoS_2_ NRR mechanism under atmospheric air, demonstrating air-to-ammonia conversion. (b) DFT simulations illustrating adsorption energy differences for strain-induced N_2_ reorientation on the V2s-MoS_2_ surface, favoring a side-on configuration under tensile strain. (c) Strain weakens the N≡N bond, elongates it, and lowers the dissociation energy barrier by enhancing electron backdonation. (d) NH_3_ evolution rate comparison of MoS_2_-3hr with reported piezocatalytic and photocatalytic NRR systems. SEM images of (e) pristine and (f) V2s-MoS_2_ NFs. (g) HRTEM image of pristine MoS_2_ showing highly active sites. STEM images of (h) MoS_2_-1hr, (i) MoS_2_-2hr, (j) MoS_2_-3hr, (k) MoS_2_-3hr with V2s vacancies, (l) MoS_2_-4hr, and (m) MoS_2_-5hr.

This is the first method to achieve high-efficiency NH_3_ synthesis solely through piezocatalysis, providing a revolutionary alternative to conventional processes that require external power and harsh conditions. By converting atmospheric N_2_ directly into NH_3_ using mechanical forces and only water, our work opens a new path for sustainable and environmentally friendly NH_3_ production. This innovation not only overcomes the limitations of current electrocatalytics for the preparation of electrodes and photocatalytic methods, such as light attenuation and rapid recombination of photogenerated carriers, but also establishes a new benchmark in green chemistry and renewable energy.

## 2 Results and Discussion

### 2.1 Catalyst Characterization

This study aims to investigate how sulfur vacancies affect the piezocatalytic performance of the NRR in water under an air atmosphere containing N_2_ gas. Variations were achieved by annealing MoS_2_ under an Ar/H_2_ gas mixture for varying durations (1 to 5 h) to create sulfur vacancies, designated as MoS_2_-*x*hr, where *x* represents the annealing time in hours. Given that MoS_2_-3hr exhibits optimal NRR performance, Figure 1e,f presents scanning electron microscopy (SEM) images of the pristine MoS_2_ NFs and MoS_2_-3hr, respectively. Figure 1g illustrates that the pristine MoS_2_ NFs feature highly single- and few-layered active sites. Additional SEM images showing varying concentrations of sulfur vacancies, controlled by different annealing durations, are provided in Figure S1 (Supporting Information). The analysis reveals that even after annealing for different durations, the MoS_2_ NFs still maintain their morphology while retaining highly active sites. The scanning transmission electron microscopy (STEM) images in Figure 1h–j reveal that annealing for 1, 2, and 3 h progressively induces the formation of sulfur vacancies. Specifically, Figure 1k reveals the presence of V2s in the 2H phase of MoS_2_, as denoted V2s-MoS_2_.^23^ These vacancies are prominently observed in the MoS_2_ annealed for 3 h (MoS_2_-3hr). As annealing time increases, individual sulfur vacancies merge to form larger pores, as shown in Figure 1l,m for MoS_2_-4hr and MoS_2_-5hr, respectively.

Figure 2a shows that the XRD diffraction peak corresponding to the 2H-phase MoS_2_ (JCPDS no. 37–1492)^24^ confirms that all samples maintain the same crystal structure. Figure S2a shows a gradual shift of the (002) plane to a lower angle, from 14.1° in MoS_2_-1hr to 13.6° in MoS_2_-5hr, along with a decrease in intensity. This shift is due to the increased S-vacancy concentration weakening the van der Waals interactions between layers, leading to an expansion in the spacing between S–Mo–S layers.^25,26^ The lattice spacing was expanded from 0.65 to 0.68 nm with increasing the annealing time from 1 to 5 h (Figure S3, Supporting Information). Figure 2b and Figure S2b show the Raman spectra of the different annealing times, with two distinguished characteristic peaks at 377 and 404 cm^–1^, corresponding to the in-plane vibration E^1^_2g_ and out-of-plane A_1g_ vibration mode, characteristic of the 2H phase. The gradual bathochromic shift and broadening observed in the E^1^_2g_ peak suggest the diminished Mo–S bonds due to the increased presence of in-plane S-vacancy numbers.^27^

**Figure 2:**
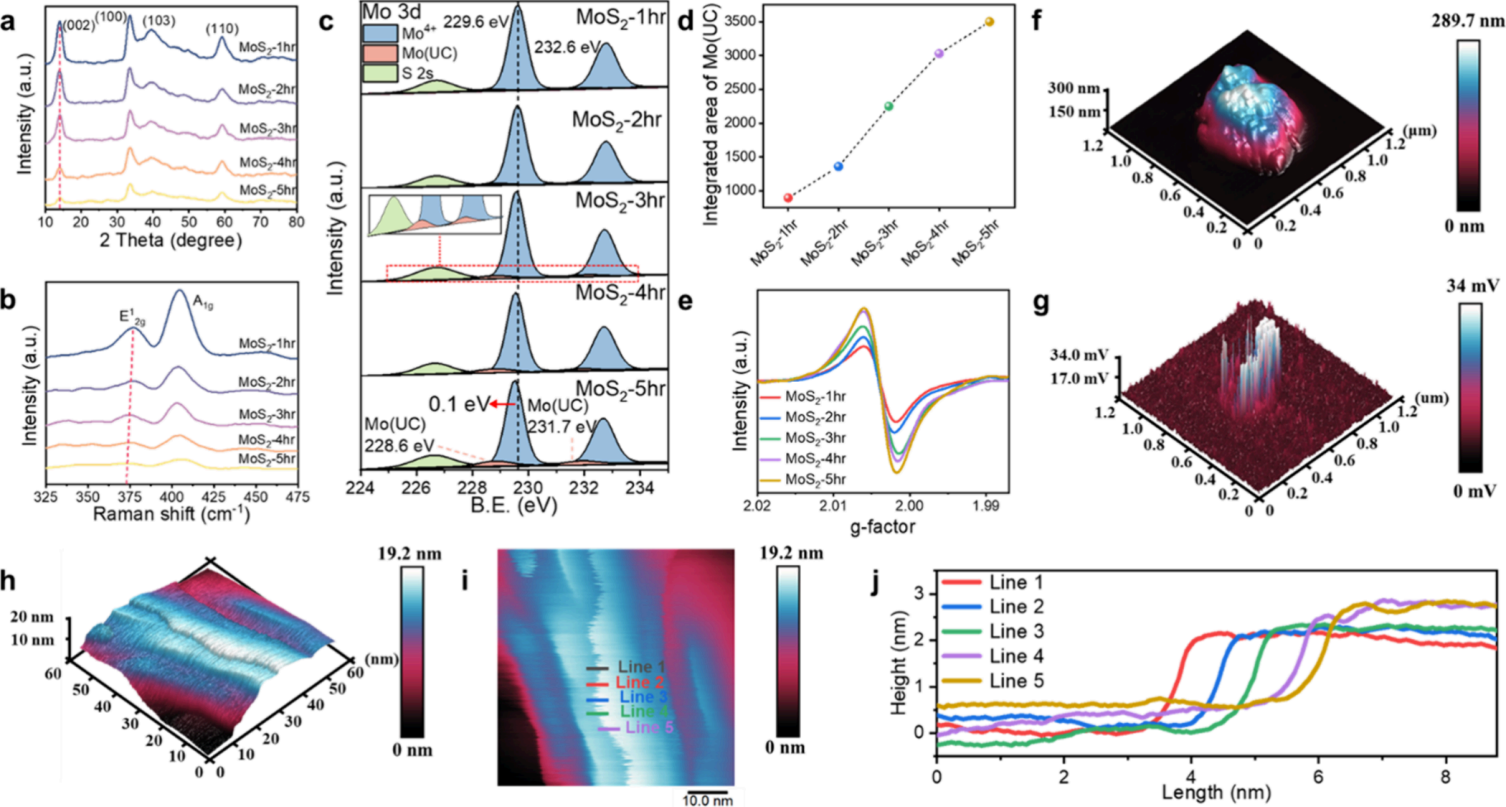
Exploring the role of defects in MoS_2_ for enhancing piezocatalytic NRR efficiency. (a) XRD pattern of V2s-MoS_2_ NFs. (b) Raman spectra of MoS_2_ after annealing for different durations, ranging from 1 to 5 h, labeled as MoS_2_-1hr to MoS_2_-5hr. (c) XPS comparison of Mo 3d spectra, revealing the appearance of the Mo (UC) peak, which gradually increases with annealing time. Inset image (MoS_2_-3hr) showing new peaks at 228.6 and 231.7 eV emerge. (d) Evolution of the Mo (UC) peak area as a function of annealing time. (e) EPR spectra of MoS_2_ NFs, illustrating changes as annealing time varies. (f) 3D topographic image of MoS_2_-3hr. (g) PFM results corresponding to the 3D topography of MoS_2_-3hr. (h) 3D topography of the magnified image at the edge sites of MoS_2_-3hr NFs (i) 2D contour map of the magnified image at the edge sites of MoS_2_-3hr NFs (j) Cross-sectional analysis of MoS_2_-3hr NFs based on the lines in (i).

XPS analysis reveals that sulfur vacancies in MoS_2_ can be controlled by adjusting the annealing times in an Ar/H_2_ atmosphere. As shown in Figure 2c, for MoS_2_-1hr, the Mo 3d spectra show peaks at 229.6 and 232.6 eV, corresponding to Mo^4+^ in the 2H phase, along with an S 2s peak. With longer annealing, new peaks at 228.6 and 231.7 eV emerge, indicating sulfur vacancies near Mo atoms (denoted as Mo (UC), inset Figure 2c, MoS_2_-3hr).^28^ The S 2p spectra show a shift of about 0.1 eV toward lower binding energy as annealing time increases, confirming the formation of S-vacancies and a rise in electron density around Mo and S atoms (Figure S2c).^25,29^ The area of the Mo (UC) peak increases with longer annealing time, showing that S-vacancies can be effectively controlled by adjusting the annealing time (Figure 2d). Electron paramagnetic resonance (EPR) spectra of Figure 2e show an increase in sulfur vacancies over time, with no large-scale defects or sulfur stripping.^30^ These vacancies expose coordinatively unsaturated Mo atoms, which are responsible for changes in the electron density and material properties. The presence of Mo (UC) peaks, along with shifts in binding energy observed in the XPS and EPR analyses, confirms the creation of these vacancies. Atomic force microscopy (AFM) was employed to analyze the thickness and corresponding piezoelectric potential (piezopotential) of the as-synthesized MoS_2_-3hr NFs and commercial MoS_2_. Figures 2f and 2g present the topographic image and corresponding piezopotential map of the MoS_2_-3hr NFs, showing a piezopotential of up to 34 mV. To provide a more comprehensive view of the surface morphology and potential distribution, the corresponding 2D images related to Figure 2f,g are included in Figure S4 (Supporting Information) for reference. The MoS_2_-3hr NFs feature numerous edge protrusions, primarily composed of stacked MoS_2_ layers. Further magnification of these edges reveals a step thickness with an average of 1.95 nm (Figure 2h,i).^31^ The cross-sectional analysis data (Figure 2j) are listed in Table S2 (Supporting Information). Extrapolating from TEM results indicating a MoS_2_ layered thickness of 0.65 nm, it can be inferred that the edge consists of approximately three layers. In contrast, commercial MoS_2_ is usually hundreds of nanometers thick (Figure S5a,b), which significantly reduces the piezopotential (Figure S5c) and leads to negligible NH_3_ production efficiency.

### 2.2 Defect Characteristics

The X-ray absorption near-edge spectroscopy (XANES) data in Figures 3a,b show a shift to lower energy levels, nearing those of metallic molybdenum (Mo^0^). This shift indicates a reduction in the Mo valence state from 4^+^ to a lower oxidation state, attributed to electron localization caused by sulfur vacancies.^29,32^ Figure 3c shows the Fourier-transform of *k* ^3^-weighted X-ray absorption fine structure (EXAFS) results, where the Mo foil standard has two peaks at 2.33 and 2.87 Å, both corresponding to Mo–Mo bonds. The second peak gives insights into the lateral size and edge relationships.^33^ The MoS_2_-1hr has two main peaks at 1.94 and 2.85 Å, corresponding to Mo–S and Mo–Mo bonds. In contrast, the MoS_2_-5hr sample shows elongation of the Mo–S and Mo–Mo bonds to 1.96 and 2.87 Å, respectively. With increasing annealing time, the Mo–S coordination decreases, indicating an increase in S-vacancy concentration, while the Mo–Mo coordination follows the same trend but decreases more gradually.^34,35^ We speculate that this discrepancy is due to the inherently edge-rich nature of MoS_2_ NFs and that the introduction of S-vacancies occurs primarily in the basal plane. This weakens the van der Waals forces between the MoS_2_ layers, which opens up the petal-like structure of the layer-to-layer stack. As a result, more edges are exposed, providing active sites for piezocatalyzing chemical reactions. The piezoresponse force microscopy (PFM) image (Figure 3d) reveals a butterfly curve that increases from MoS_2_-1hr to MoS_2_-3hr, followed by a decline to MoS_2_-5hr. This behavior indicates that the introduction of S-vacancies enhances the piezoelectric response of MoS_2_ NFs, while an excess of S-vacancies may disrupt the structural integrity, leading to the formation of defect centers and a subsequent reduction in piezoelectric performance.^36,37^ To further clarify this issue, we estimated an effective d_33_ value based on the experimentally obtained butterfly curve (Supporting Information of S1).

**Figure 3:**
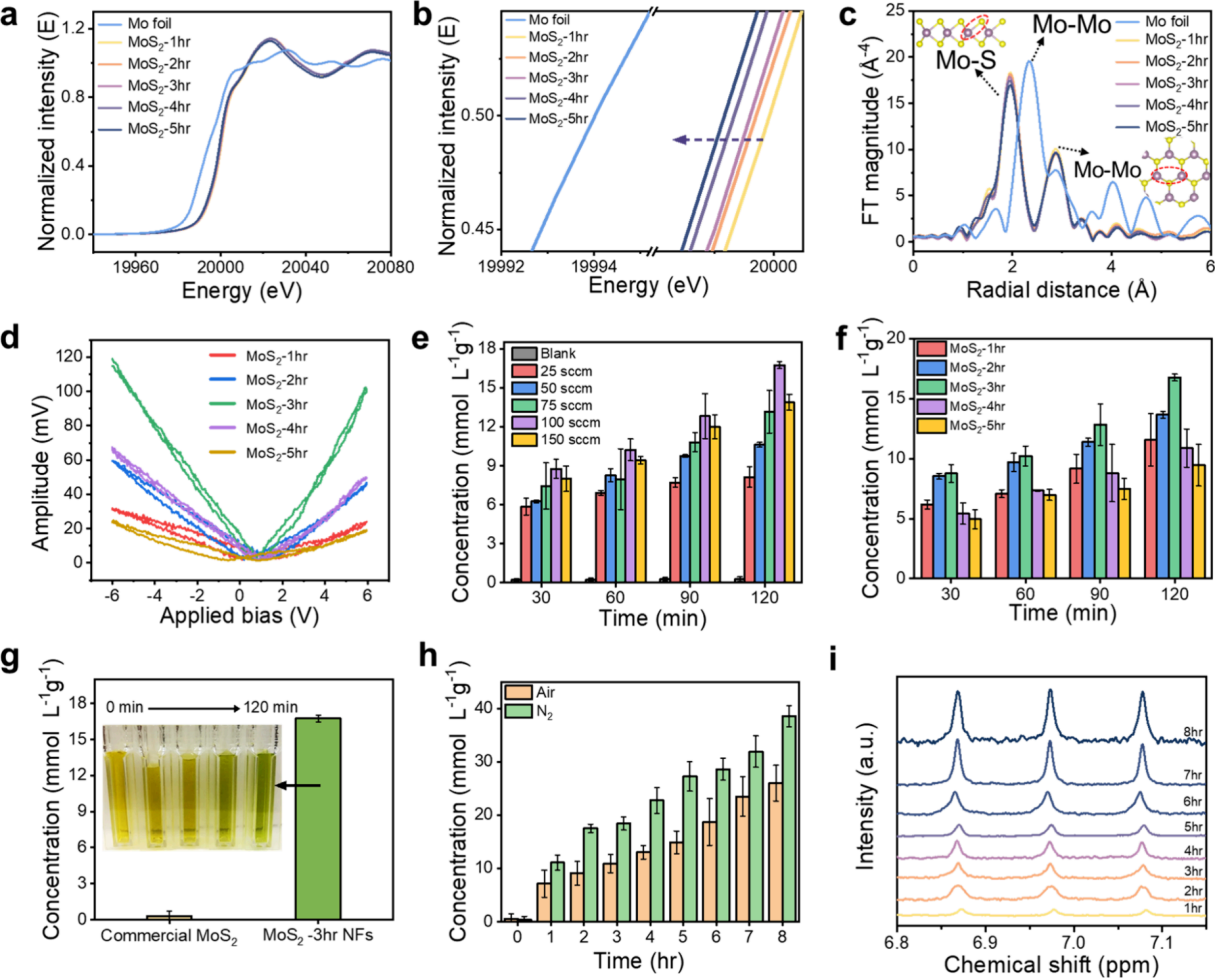
Quantitative analysis of the piezocatalytic performance of V2s-MoS_2_ piezocatalyst in NRR. (a) Normalized Mo K-edge XANES spectra and (b) a magnified view of the edge region. (c) Fourier-transform Mo K-edge EXAFS spectra. (d) Butterfly curve of all samples. (e) NH_3_ yield rate of MoS_2_-3hr under different N_2_ flow rates. (f) NH_3_ yield rate for the different annealing times of MoS_2_ NFs. (g) NH_3_ yield rate compared between bulk MoS_2_ and V2s-MoS_2_-3hr. (h) NH_3_ yield rate of MoS_2_-3hr under an 8 h long-term testing using air and N_2_ as the feeding gas. (i) corresponding NMR spectra to 8 h long-term testing using air as the feeding gas.

We conducted the piezocatalytic NRR using V2s-MoS_2_ NFs in deionized water saturated with N_2_, without sacrificial agents, and under dark conditions. The quantity of as-catalyzed NH_3_ was determined by the indophenol blue method (IPB) with its calibration curve in Figure S6 (Supporting Information). Figure 3e shows that NH_3_ formation occurs at all N_2_ flow rates during the conversion of N_2_ to NH_3_, except in the blank sample, where no catalyst was added. The small, nonzero values observed in the blank sample can be disregarded.^38^ Moreover, the yield of NH_3_ shows a trend of increasing with the flow rate, reaching 16.75 mmol L^–1^g^–1^ when the flow rate reaches 100 sccm within 2 h. Nevertheless, increasing the flow further to 150 sccm resulted in a reduced yield of 13.9 mmol L^–1^g^–1^. This reduction is mainly due to excessive flow rates forming large bubbles that burst, pushing the catalyst to the reactor walls and preventing it from participating in the reaction. As depicted in Figure 3f, the MoS_2_ NFs display a substantial NRR enhancement with increasing S-vacancy concentrations. Increasing the V2s concentration from MoS_2_-1hr to MoS_2_-5hr resulted in a significant improvement in NH_3_ yield. The optimal yield of 16.75 mmol L^–1^g^–1^ was achieved with MoS_2_-3hr (∼8.4 mmol L^–1^g^–1^h^–1^). This enhancement can be attributed to the reinforced piezoelectric polarization facilitated by a moderate concentration of S-vacancies. As the concentration of S-vacancies increased, NH_3_ production steadily declined, reaching its lowest level in the MoS_2_-5hr sample (9.45 mmol L^–1^g^–1^), indicating that excessive S-vacancies further degraded the piezoelectric properties.^36^ The commercial MoS_2_ had a thicker structure, which hindered its intrinsic piezoelectric characteristics and,^39^ as a result, diminished its NRR performance, as shown in Figure 3g. To further verify that the generated ammonia originates from the externally supplied nitrogen gas, we conducted a control experiment using argon as the feed gas instead of N_2_, while maintaining the same flow rate of 100 sccm and the same reaction conditions. As shown in Figure S7, when Ar was used as the reaction atmosphere, the detected NH_3_ concentration was extremely low and comparable to that of the blank sample, with no significant ammonia formation observed. However, when N_2_-saturated DI water was used under an Ar flow, a small amount of ammonia was initially detected. Importantly, this value did not increase over time, indicating that the ammonia was generated only from limited dissolved N_2_ in the water. Once this dissolved N_2_ was depleted, no further NRR occurred due to the lack of a continuous nitrogen gas supply. Additionally, this finding also helps explain the nonlinear behavior observed between the early stage (0–30 min) and the subsequent time intervals—during the early stage, a sufficient concentration of dissolved N_2_ supports a stable reaction rate; however, without sufficient gas exchange, the N_2_ becomes depleted over time, leading to a slower reaction rate and deviation from ideal linear behavior. This result strongly supports that the ammonia generation in this study arises from the piezocatalytic reduction of molecular N_2_ by the catalyst rather than from background contamination or other artifacts.

To demonstrate the practical potential of piezocatalysis for N_2_ reduction, we successfully used N_2_ from air in water, marking a significant breakthrough. As illustrated in Figure 3h, the V2s-MoS_2_ NFs under continuous ultrasonic vibration led to a remarkable accumulation of NH_3_, reaching 26.03 mmol L^–1^g^–1^ (equivalent to an absolute yield of 2.603 μmol) after 8 h with air and 36.55 mmol L^–1^g^–1^ (equivalent to an absolute yield of 3.655 μmol) with N_2_ as feeding gas. The yield with N_2_ as the feed gas was 1.46 times higher than with air, as evidenced by the color changes in Figure S8a (IPB colorimetric analysis) and the UV–vis spectra in Figure S8b,c (Supporting Information S2), which aligns with a previous study reporting reduced performance when switching from N_2_ to air, primarily due to the lower solubility of N_2_ in water.^16^ Despite the presence of oxygen, the observed ammonia production remained high. This can be attributed to two key factors: (1) all experiments were conducted using DI water that had been presaturated with ultrahigh-purity N_2_, effectively minimizing dissolved oxygen and enriching N_2_ availability; and (2) due to the inherently low solubility of gases in water, the replacement of dissolved N_2_ by O_2_ during the air-feeding reaction is kinetically limited. As a result, the dissolved N_2_ remains sufficient to support sustained NRR activity, even after switching to air. The corresponding ^1^H NMR analysis results from the IPB method, shown in Figure 3i, are in excellent agreement, confirming the accuracy of the NH_3_ measurements. This reliable NH_3_ production from air marks a significant breakthrough in renewable energy and nitrogen fixation achieved without high temperatures, pressures, or sacrificial agents. Moreover, to ensure that the observed catalytic performance is not accompanied by degradation of the active sites or structural instability, a series of comprehensive characterizations was conducted on the MoS_2_-3hr catalyst before and after the piezocatalytic NRR process, as shown in Figure S9 (Supporting Information S3). In particular, XPS analysis of the N 1s region (Figure S10a,b) revealed no emergence of nitrogen-containing species after annealing and catalysis. The absence of detectable peaks corresponding to residual Mo–N bonds or adsorbed nitrogen-based compounds confirms that the observed NH_3_ production does not originate from nitrogen impurities or precursor residues, but rather from the reduction of externally supplied N_2_. These results collectively demonstrate that the V2s-MoS_2_ catalyst not only is highly effective in driving nitrogen reduction under ambient conditions using either N_2_ or air but also exhibits excellent structural and chemical stability, maintaining its active sites and morphology throughout the piezocatalytic process.

To identify the N_2_ source used in converting N_2_ to NH_3_, we conducted an isotopic labeling study with ^15^N as the purging gas and analyzed the results using NMR. Figure 4a shows that when ^14^N_2_ was used as the purging N_2_ gas, we observed distinct triple peaks corresponding to ^14^NH_4_^+^, with a coupling constant of J_14N–H_ = 52 Hz, which matched the reference spectra of ^14^NH_4_Cl. Upon switching to ^15^N_2_, a clear doublet peak with a coupling constant of J_15N–H_ = 72 Hz attributed to ^15^NH_4_^+^ was evident in the spectrum, accompanied by a minor presence of the triple peaks corresponding to ^14^NH_4_^+^.^40,41^ Compared to the blank sample (without catalyst), no ^14^N_2_ signal was observed in a saturated ^14^N_2_ pure water solution. However, our MoS_2_ NFs not only efficiently catalyze the conversion of ^14^N_2_ from the environment but also convert ^15^N_2_ into NH_3_ (Figure 4a). Due to the extremely low concentration of ^15^N_2_ in the environment, the presence of ^15^NH_4_^+^ further confirms that piezocatalysis effectively reduces N_2_ atoms to NH_3_. In addition, because a nitrogen-containing precursor (thiourea) was used during the synthesis of the MoS_2_ NFs, we conducted a 7-day soaking test to evaluate the possibility of ammonia contamination originating from the catalyst surface, as shown in Figure S11 (Supporting Information of S4).

**Figure 4:**
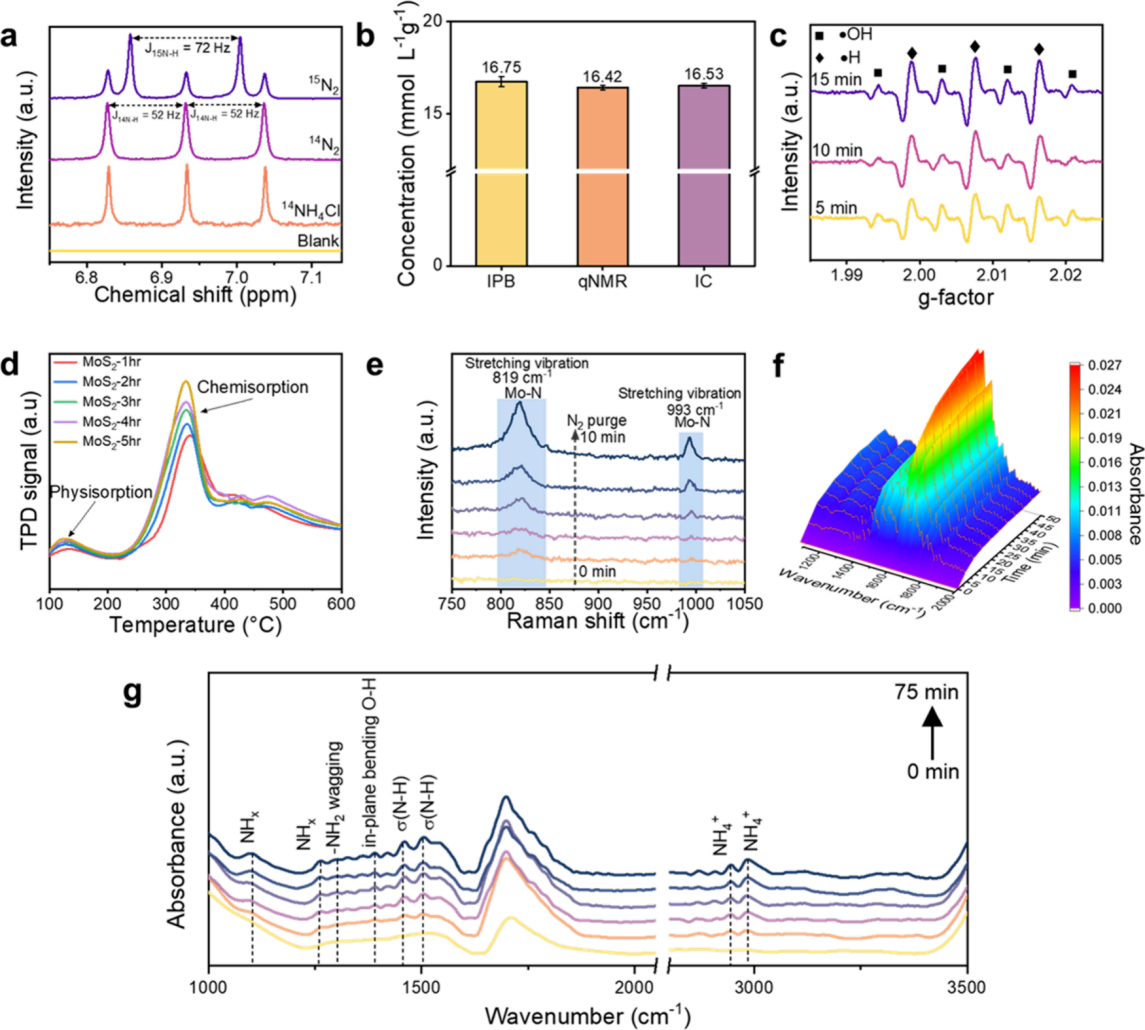
Investigation of active sites and reaction mechanism for NRR. (a) ^1^H NMR spectra of the blank test and MoS_2_-3hr using ^14^N_2_ and isotope ^15^N_2_ as feeding gases, with a reference spectrum of ^14^NH_4_Cl. (b) NH_3_ yield rates of MoS_2_-3hr, measured by IPB, qNMR, and IC methods. (c) EPR spectra of MoS_2_-3hr, showing the generation of hydrogen radicals. (d) N_2_-TPD profiles of MoS_2_ NFs at different annealing times. (e) In situ Raman spectra of MoS_2_-3hr following N_2_ adsorption. (f) In situ DRIFT spectra of MoS_2_-3hr during N_2_ adsorption. (g) Liquid-phase in situ DRIFTS for real-time analysis of reaction mechanisms, highlighting the importance of intermediate states in the evolution of key reaction stages.

To account for the potential susceptibility of the IPB method to environmental interference,^38^ additional NH_3_ detection methods were employed using MoS_2_-3hr as a benchmark. These methods included quantitative nuclear magnetic resonance (qNMR) and ion chromatography (IC),^7,42^ with their calibration curves provided in Figures S12 (Supporting Information S5) and S13 (Supporting Information S6). Results from IPB, qNMR, and IC were consistent, confirming reliable NH_3_ production (Figure 4b). Additionally, tests for byproducts such as N_2_H_4_ and NO_*x*_ were conducted using the Watt-Chrisp method and anion IC.^42,43^ No N_2_H_4_ was detected (Figure S14, Supporting Information S7), and no NO_*x*_ anions were produced before or after the reaction (Figure S15, Supporting Information S8), demonstrating high selectivity for NH_3_ production. The *R*-squared values for all measurements exceed 0.997.

A pivotal step in the N_2_ reduction to NH_3_ reaction is the hydrogenation reaction. The piezoelectric polarization induced by S-vacancies in MoS_2_ under strain conditions could enhance the transfer of electrons, resulting in the generation of abundant hydrogen radicals (•H).^40,42^ These radicals actively participate in and promote the hydrogenation process and can be directly observed through EPR with the assistance of a trapping agent (5,5-dimethyl-1-pyrroline-N-oxide; DMPO). In Figure 4c, the EPR results reveal seven characteristic signals that stem from two types of radicals: three signals contributing to •H exhibit higher intensities, while the remaining four signals with lower intensities originate from hydroxyl radicals (•OH). Furthermore, stronger signals are detected with increasing ultrasonic times, indicating an increase in the concentration of •H. This enhancement is attributed to the tendency of free radicals generated by the piezoelectric response triggered by the rupture of bubbles to react with DMPO and form DMPO–H and DMPO–OH.^44^ This observation suggests that the •H generation reaction can continue over time, thereby continuously providing the resources required for the hydrogenation reaction to form NH_3_. The sulfur vacancies not only alter the electronic structure and piezoelectricity of MoS_2_ but also confer different surface characteristics to the material, depending on the varying concentrations of sulfur vacancies. The N_2_ temperature-programmed desorption (TPD) profile of MoS_2_ NFs with various S-vacancy concentrations (Figure 4d) provides compelling evidence: as the vacancy concentrations increase, the chemisorption capacity of MoS_2_ for N_2_ also increases. With a higher N_2_ adsorption capacity enabled by modulating the S-vacancy concentration, more pathways are paved for electrons to undergo the reduction reaction efficiently. A Brunauer–Emmett–Teller (BET) surface area analysis was conducted, and the results in Figure S16 indicate that MoS_2_ NFs with different S-vacancy concentrations possess nearly identical specific areas, suggesting that the variations in N_2_ adsorption obtained from the TPD test are attributed to the concentration of vacancies. Figure 4e shows the in situ Raman spectra of MoS_2_-3hr purged with N_2_ gas. With increasing reaction time, two distinct peaks located at approximately 819 and 993 cm^–1^ correspond to Mo–N stretching vibrations and gradually intensify.^45^ Figure 4f shows that the in situ diffuse reflectance infrared Fourier-transform spectroscopy (DRIFT) further provides the adsorption configuration of N_2_ molecules, demonstrating that a peak at 1650 cm^–1^ corresponds to a side-on configuration, exhibiting an increasing trend over time.^46,47^ Results from XPS, Raman, and DRIFT analyses indicate that unsaturated Mo atoms exposed by sulfur vacancies act as active sites for nitrogen bonding.

Liquid-phase in situ DRIFTS enables real-time analysis of reaction mechanisms, providing critical insights during key reaction stages. As shown in Figure 4g, the peaks observed at 2987 and 2940 cm^–1^ were attributed to the NH_4_^+^ produced by MoS_2._ The peaks observed at 1456 and 1497 cm^–1^ could be attributed to the σ­(N–H) bending mode derived from the intermediate (i.e., NH_*x*_ and N_2_H_*y*_) and the NH_3_. Similarly, the peak observed at 1304 cm^–1^ corresponds to the wagging mode of −NH_2_, while the peaks observed at 1097 and 1262 cm^–1^ could preliminarily be assigned to the NH_*x*_ intermediate.^48–51^ Additionally, the peak at 1389 cm^–1^ is ascribed to the in-plane bending vibration of the activated O–H bond. The results show gradual hydrogenation of nitrogen to NH_3_ under piezocatalysis, highlighting potential intermediates for analysis via DFT simulation.

### 2.3 DFT Simulation

Scanning transmission electron image results showed that V2s vacancies on the MoS_2_ basal plane could serve as active sites for N_2_ fixation. DFT calculations were conducted to determine the N_2_ adsorption energy across various configurations and to evaluate the effects of strain on charge transfer, Mo–N/N-N bonding, and the N_2_ reduction mechanism at the active site. Figure 5a depicts the defect model of V2s-MoS_2_ while Figure S17 represents the dumbbell-shaped N_2_ molecules in three distinct adsorption configurations: one end-on configuration and two different side-on configurations. In Figure S17 (Supporting Information of Figure S9), we analyzed N_2_ adsorption in both side-on and end-on configurations under strains of −10, −5, 0, +5, and +10% (with negative values indicating compressive strain and positive values indicating tensile strain). The results show that N_2_ molecules preferentially adopt a side-on configuration over an end-on configuration when subjected to tensile strain. Figure 5b shows that as the strain increases from 0 to +5 and +10%, the adsorption energies of N_2_ in the side-on configuration decrease from +0.05 to −1.47 and −3.51 eV, respectively. This trend suggests that tensile strain can increase the adsorption of nonpolar N_2_ molecules onto the MoS_2_ surface, thereby facilitating the N_2_ reduction process. Conversely, the adsorption energy increases for both the side-on and end-on configurations under compressive strain, indicating that compressive strain results in diminished N_2_ fixation ability on V2s-MoS_2_. Figure 5c shows that the relationship between adsorbed N_2_ bond length and strain reveals that the side-on N_2_ bond length increases with the escalation of tensile strain, which can be evident for the N≡N triple bond weakening with tensile strain engineering. In contrast, Figure S18a,b shows the simulation results for a single sulfur vacancy site. The adsorption energy of N_2_ on a single sulfur vacancy remains positive under both unstrained and compressive strain conditions, indicating that the adsorption is thermodynamically unfavorable. Even under tensile strain, N_2_ exhibits only weak physical adsorption on the single vacancy site, with relatively low adsorption energy and negligible bond elongation. This suggests that the single sulfur vacancy site offers poor potential for NRR activity. In summary, these comparative results clearly demonstrate that dual sulfur vacancies provide a more favorable chemical environment for N_2_ activation than single vacancies, in terms of both stronger adsorption and more effective bond activation under strain engineering.

**Figure 5:**
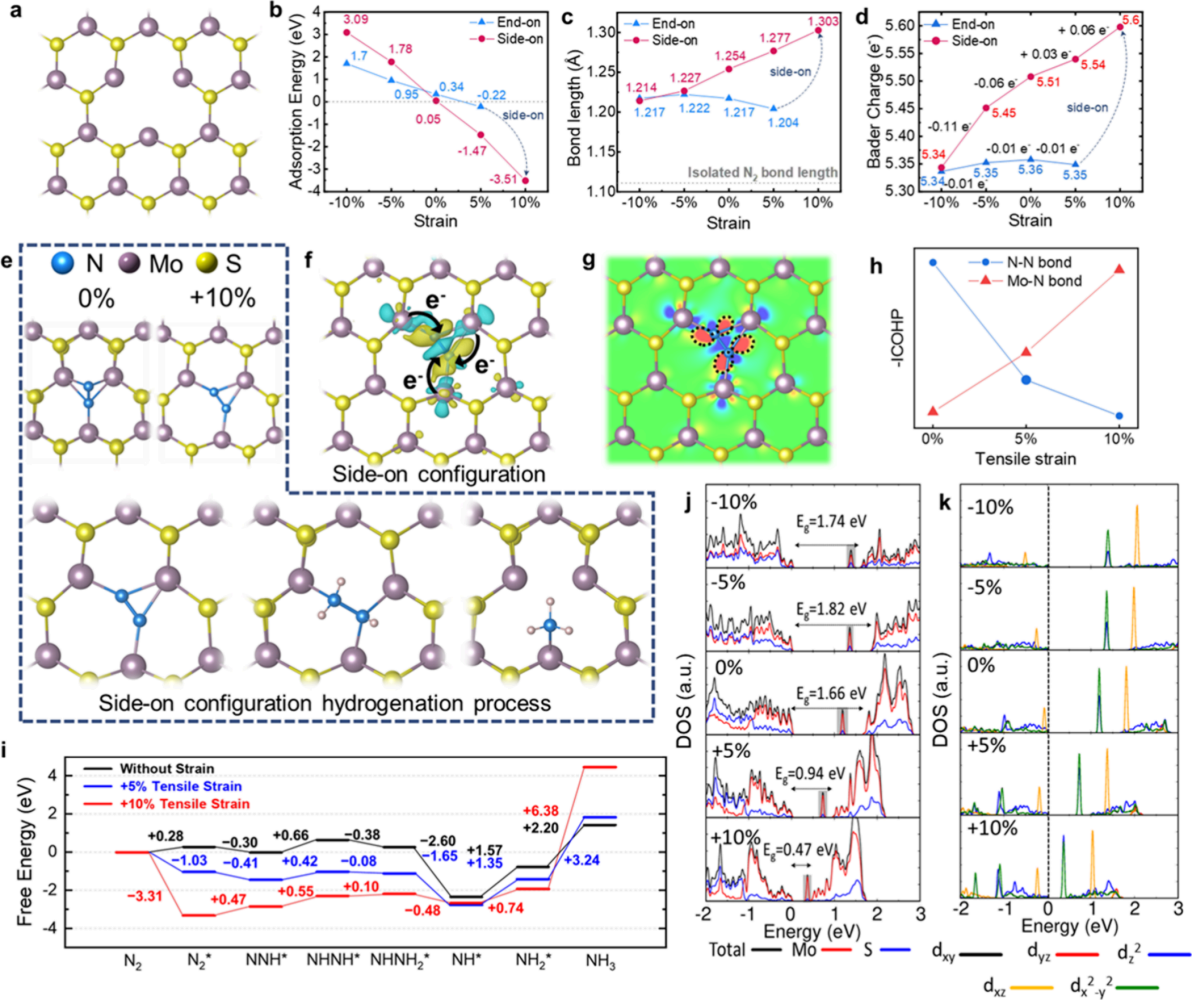
DFT studies of the reaction mechanism of NRR by defective MoS_2_. (a) Top view of the geometric structure of monolayer V2s-MoS_2_. (b) Effect of compressive and tensile strain on the adsorption energy of V2s-MoS_2_. (b) Effect of compressive and tensile strain on the adsorption energy of V2s-MoS_2_. (c) Relationship between N_2_ bond length and adsorption on V2s-MoS_2_ under compressive and tensile strain. (d) Relationship between the average Bader charge of N_2_ and its adsorption on V2s-MoS_2_ under compressive and tensile strain. The blue dashed line represents the change of end-on transition to the side-on configuration’s condition. (e) N_2_ molecules were adsorbed in a side-on configuration on V2s-MoS_2_ under both unstrained conditions (0%) and with +10% tensile strain. The gray, yellow, and blue balls are Mo, S, and N atoms, respectively. The bottom panel illustrates the stepwise hydrogenation process of N_2_ molecules adsorbed in a side-on configuration on the MoS_2_ surface. (f) Charge density difference of N_2_ adsorbed on V2s-MoS_2_ with +10% tensile strain represents the electron accumulation and depletion within yellow and cyan, respectively. (g) Contour plot of the (f), the red and blue regimes represent the charge accumulation and depletion, respectively. (h) ICOHP plot of bond strength analysis for Mo–N and N≡N. (i) Free energy profiles for NH_3_ production on MoS_2_ under three conditions: strain free (black), +5% tensile strain (blue), and +10% tensile strain (red). Tensile strain lowers the activation barrier for N_2_ and key hydrogenation steps, with +5% strain showing the most thermodynamically favorable overall pathway. (j) Total DOS of V2s-MoS_2_ under different percentages of strain (k) PDOS of first-neighbor Mo near S-vacancy of V2s-MoS_2_ under different percentages of strain.

The Bader charge method is employed to quantitatively evaluate the charge transferred per N_2_ atom. As shown in Figure 5d, without any strain, N_2_ in a side-on configuration adsorbed on V2s-MoS_2_ gains a charge of +0.51 (increasing from 5 to 5.51 compared with a single nitrogen atom, which has five valence electrons). In contrast, the end-on configuration gains a smaller charge of +0.36 (from 5 to 5.36). Furthermore, as the tensile strain increases from 0 to +5 and +10%, the Bader charge rises from 5.51 to 5.54 and 5.60, respectively, indicating that N_2_ gains an additional 0.03 e^–^ from 0 to +5% strain and 0.06 e^–^ from +5 to +10% strain. This suggests that the tensile strain can facilitate the electron transfer from V2s-MoS_2_ to N_2_, which benefits the NRR. Conversely, in a side-on configuration under compressive strain, the transferred charges decrease from 5.51 to 5.45 at −5% strain and further to 5.34 at −10% strain. However, this remains higher than the number of electrons obtained by N_2_ in the end-on configuration in either scenario. In the end-on configuration, minimal electron transfer to N_2_ occurs, making it unfavorable for the reduction reaction. It should be noted that the analysis of adsorption energy, bond length, and Bader charge shows that the end-on configuration becomes unstable under +10% strain, ultimately transitioning to the side-on configuration (Figure 5b–d). The evidence indicates that N_2_ molecules adsorbed on the surface of V2s-MoS_2_ in a side-on configuration exhibit the most favorable adsorption condition observed in our calculations (Figure 5e).

To further explore the electron transfer dynamics between V2s-MoS_2_ and side-on adsorbed N_2_ under +10% strain, the charge density difference (CDD) was computed, as shown in Figure 5f. The presence of sulfur vacancies leads to Mo atoms neighboring the vacancies possessing undersaturated 3*d* orbitals, facilitating the electron backdonation into the antibonding π* orbitals of the adsorbed N_2_. The CDD highlights the electron-rich local Mo atoms adjacent to sulfur vacancies, through which electrons are injected into the side-on adsorbed N_2_. Additionally, the contour plot of the CDD (Figure 5g) depicts electron accumulation around the N_2_ molecule. By accepting electrons into antibonding π* orbitals, the strong orbital interaction between Mo and N decreases the bonding strength and the activation barrier for N_2_ reduction. Moreover, the charge depletion observed at the central region of the N–N bond suggests attenuation of the triple bond in N_2_ molecules. The integrated crystal orbital Hamilton population (ICOHP) was used to study how tensile strain on MoS_2_ affects N_2_ molecule adsorption. −ICOHP analysis provides insight into bond strength by examining the overlap of the atomic orbitals involved in bonding. A higher −ICOHP value indicates a greater degree of orbital overlap, reflecting stronger bonding strength. As shown in Figure 5h, the increase in tensile strain results in N_2_ receiving more electrons, weakening the N–N bond, while the Mo–N interaction strengthens with the application of greater tensile strain, favoring the adsorption of N_2_ molecules.

The piezocatalytic hydrogenation process likely follows an enzymatic mechanism rather than a distal or alternating one. Under tensile strain, N_2_ molecules prefer a side-on configuration on V2s-MoS_2_, which leads to hydrogenation and NH_3_ production, as confirmed by our calculations (Figure 5b–d), rather than an end-on configuration. The strain-dependent hydrogenation process on V2s-MoS_2_ involves a series of intermediate steps, with each state exhibiting distinct free energy changes under different strain conditions (Figure 5i). This free energy diagram reveals that tensile strain markedly influences N_2_ activation and subsequent hydrogenation steps. Without strain, N_2_ activation (N_2_ → N_2_*) is thermodynamically unfavorable, whereas +5 and +10% tensile strain lower the free energy by −1.03 and −3.31 eV, respectively. The first hydrogenation step (N_2_* → NNH*) is facilitated under +5% strain (−0.41 eV), but becomes unfavorable at +10% strain (+0.47 eV). Subsequent hydrogenation steps, NNH* → NHNH* and NHNH* → NHNH_2_*, are also promoted under +5% strain. These results indicate that an appropriate level of tensile strain can effectively enhance the hydrogenation process. These results suggest that tensile strain not only enhances N_2_ activation but also promotes the reaction through intermediate states such as NNH*, NNH_2_*, and NH*. In the final stages, when transitioning from NH_2_* to NH_3_, the free energy under +10% strain shows a notable increase, reaching a maximum value of +6.38 eV. This rise in free energy reveals that the formed product of NH_3_ will not stably adsorb on the MoS_2_ and will instead diffuse away, aligning with experimental observations. The DFT calculation suggests that tensile strain engineering can not only raise the capability of N_2_ reduction and promote charge transfer on V2s-MoS_2_ but also significantly boost the activation of N_2_ and decrease the energy barrier. According to the charge transfer result (Figure 5f), charge transfer primarily occurs between Mo and N atoms.

To examine the changes in the electronic structure under applied strain and to understand how Mo atoms interact with N_2_ molecules, the total density of state (DOS) and projected density of states (PDOS) for atoms in V2s-MoS_2_ are presented in Figure 5j,k, respectively. It is important to note that the PDOS plots focus only on the three Mo atoms, which are the first neighbors to the V2s vacancies. Figure 5j shows that the introduction of V2s defect states (see the peak in the shaded gray area) slightly reduces the band gap by 0.06 eV compared to defect-free MoS_2_ NFs without strain (Figure S19). It also reveals that tensile strain can significantly narrow the band gap of MoS_2_ from 1.66 to 0.94 (+5% strain) and 0.47 eV (+10%). This band gap reduction implies that electrons are more easily excited from the valence band to the conduction band, where the hot electron in the conduction band can be harnessed to reduce the N_2_ molecule. The Mo *d* orbitals consist of five suborbitals: *d*_*xy*_, *d*_*xz*_, *d*_*yz*_, *d*_*x*_^2^_*‑y*_^2^, and *d*_*z*_^2^, each with distinct shapes and orientations, enabling complex bonding interactions. These suborbitals are crucial in determining the chemical behavior and bonding characteristics of transition metals. Figure 5k reveals that the existence of sulfur vacancies impacts the electronic structures of Mo atoms in two key ways. First, the formation of V2s vacancies introduces occupied *d*_*xz*_ orbitals, which exhibit a four-lobe shape. The symmetry of the wave function closely aligns with the LUMO of N_2_, leading to significant geometric overlap. This wave function overlap facilitates orbital hybridization, which favors the side-on adsorption configuration of N_2_ on V2s-MoS_2_ over the end-on configuration. Second, the V2s vacancies generate empty intermediate states, primarily derived from *d*_*x*_^2^_*–y*_^2^, and *d*_*z*_^2^ orbitals, within the band gap of V2s-MoS_2_, acting as electron-trapping states that extend carrier lifetime. Accordingly, both strain and sulfur vacancies play essential roles in modifying surface properties, significantly influencing the electronic and catalytic behavior of MoS_2_. This discovery of S-deficient MoS_2_ piezocatalysts introduces a groundbreaking and efficient method for NH_3_ synthesis directly from atmospheric N_2_ without the need for sacrificial agents, showcasing the potential of piezocatalytic systems in advancing sustainable energy solutions. In addition to high ammonia yields, the MoS_2_-3hr catalyst exhibited excellent selectivity toward the NRR. Based on gas chromatography (GC) analysis, the H_2_ evolution rate was measured to be 11.32 μmol g^–1^ h^–1^ (Figure S20a,b), compared to an NH_3_ production rate of 83.75 μmol g^–1^ h^–1^, corresponding to an NRR selectivity of 88.1% (Figure S20c). This high selectivity highlights the suppression of the competing hydrogen evolution reaction, which is a common challenge in aqueous NRR systems. Furthermore, O_2_ generation was detected during the reaction, indicating that water oxidation is the likely oxidation half-reaction that balances the electron consumption from NRR (Supporting Information S10). These results collectively demonstrate that the MoS_2_-3hr catalyst not only promotes efficient and selective NH_3_ formation but also maintains a favorable redox balance under piezocatalytic conditions.

## 3 Conclusions

This study introduces a groundbreaking approach to atmospheric NRR using sulfur-deficient MoS_2_ nanoflowers as piezocatalysts, achieving a noteworthy NH_3_ yield of 8374.8 ± 140.1 μmol L^–1^ g^–1^ h^–1^ corresponding to an absolute production rate of 0.84 ± 0.01 μmol h^–1^ in nitrogen-saturated water without the need for sacrificial agents among piezocatalytic systems. In an 8 h test, our method achieved an NH_3_ production rate of 36.55 mmol L^–1^g^–1^ (equivalent to an absolute yield of 3.655 μmol). Remarkably, when air was used as the feeding gas, the catalyst still performed efficiently, producing NH_3_ at 26.03 mmol L^–1^g^–1^. This performance not only surpasses the capabilities of current photocatalysts and piezocatalysts but also rivals the efficiency of top-tier electrocatalysts, establishing a new benchmark in the field of nitrogen fixation. The creation of double sulfur vacancies (V2s) significantly enhances the adsorption and activation of N_2_ molecules with the piezoelectric response of MoS_2_, facilitating their dissociation under mild conditions. The mechanical strain applied to the MoS_2_ NFs generates a piezoelectric potential that effectively separates electron–hole pairs, driving the reduction reaction with unparalleled efficiency. DFT results revealed that the sulfur vacancies in MoS_2_ alter the electronic structure, enabling a strong interaction between the unpaired Mo *d* orbital electrons and the π* antibonding orbitals of N_2_. This interaction leads to weakening of the N≡N triple bond, elongating its bond length and lowering the energy barrier for dissociation. The results demonstrate that tensile strain further enhances this process, facilitating more efficient electron transfer and nitrogen reduction. Importantly, gas chromatography analysis revealed a high NRR selectivity of 88.1%, based on the ratio of NH_3_ to total gaseous products (NH_3_ + H_2_), with only a small amount of H_2_ evolution observed. In addition, O_2_ production was confirmed as a result of water oxidation, balancing the overall redox reaction. These findings underscore the catalyst’s strong preference for N_2_ reduction over competing side reactions, even under ambient conditions. The integration of sulfur vacancy engineering with piezoelectric effects in MoS_2_ NFs highlights piezocatalysis as an efficient and sustainable method for NH_3_ synthesis, opening up new possibilities for future innovations.

## 4 Methods

### Synthesis of MoS_2_ NFs

The synthesis of 2H MoS_2_ NFs involved a hydrothermal process. Initially, 0.7 g of thiourea (CH_4_N_2_S, 99%; Alfa Aesar) and 0.7 g of sodium molybdate dihydrate (Na_2_MoO_4_·2H_2_O, 99%; Alfa Aesar) were dissolved in a 100 mL beaker containing 30.0 mL of deionized (DI) water and mixed thoroughly by magnetic stirring to achieve a homogeneous solution. Subsequently, 1 mL of 1 M 1-butyl-3-methylimidazolium chloride (BMIM [Cl, 99%; Sigma-Aldrich) was added to the solution. A diluted hydrochloric acid solution was prepared by mixing 1 mL of concentrated hydrochloric acid (HCl, ACS Reagent, 36.5–38.0%; Honeywell) with 28 mL of DI water to adjust the solution’s pH. After stirring for 30 min, the resulting homogeneous solution was transferred to a 100 mL stainless autoclave, heated to 220 °C, and maintained for 24 h. Following the heating process, the black powder obtained was washed several times with DI water and ethanol (EtOH, 99.99%; Honeywell) through centrifugation and subsequently dried at 70 °C for 12 h in a vacuum oven.

### Synthesis of V2s-MoS_2_ NFs

The 0.2 g of prepared 2H MoS_2_ NFs were placed into a tube furnace filled with Ar/H_2_ (95:5, purity: 99.99%) atmosphere at a flow rate of 100 sccm.^52^ The temperature was ramped up to 300 °C at a rate of 10 °C/min, followed by different holding times (1 to 5 h). The resulting V2s-MoS_2_ NFs obtained under varied holding times were labeled as MoS_2_-*x*hr, where *x* ranged from 1 to 5.

### NRR Reaction

The NRR experiment involves using 10 mL of deionized (DI) water, which has been saturated with high-purity N_2_ gas (99.999%) overnight, and 10 mg of presynthesized V2s-MoS_2_ NFs catalysts. The entire reaction system is contained in a 25 mL glass reaction vessel. The mixture undergoes ultrasonic oscillation (SB-300DTY, 300W, 40 kHz) to provide mechanical force and initiate piezocatalyzing. During the ultrasonication, the system continuously pumps with N_2_, purified using a 0.01 M H_2_SO_4_ solution in a gas purification system to eliminate any possible NH_3_ or nitrogen oxide (NO_*x*_) contaminants,^32,53^ and then the gas was continuously fed into the sample bottle at a controlled flow rate (0–100 sccm) through a mass flow controller. To monitor ammonia production, the indophenol blue method was employed using a UV–visible spectrophotometer (Hitachi U-3900). A 1 mL portion of the reaction solution was extracted every 30 min during the catalytic process. The color reagent was prepared by dissolving 4.97 g of salicylic acid and 4.39 g of sodium citrate in 100 mL of a 0.625 mol/L KOH aqueous solution. After adding 1.25 mL of the color reagent, 150 μL of 10 mg/mL sodium nitroferricyanide (C_5_FeN_6_Na_2_O) solution, and 75 μL of sodium hypochlorite (NaClO, available chlorine 4.0%), the mixture was left to stand at room temperature for 2 h. Finally, the absorbance at 658 nm was measured to determine the concentration of NH_3_.

For the atmospheric N_2_ reduction experiments, all reaction conditions remained identical to those of the N_2_ gas experiments except that air was used as the feeding gas instead of high-purity nitrogen.

### Material Characterization

Field-emission SEM (SU8010; Hitachi), high-resolution TEM (JEM-F200; JEOL), and AFM (Dimension Icon; Bruker) were used to examine the morphologies of the synthesized MoS_2_ NFs. PFM was used to determine the material’s piezoelectric properties. XRD (D2 Phaser; Bruker) was used to characterize the crystallographic structure of MoS_2_. XPS and Auger electron spectroscopy (ESCALAB Xi+; Thermo Fisher Scientific) were performed to compare the chemical states of different annealing times for MoS_2_. EPR (Elexsys-E580 series; Bruker) was conducted to evaluate the formation of S and obtain signals of hydrogen radicals in catalysis reactions. UV–visible spectroscopy (U-3900; Hitachi) was employed to quantitatively estimate the concentration of NH_3_. Ion chromatography (Dionex Integrion; Thermal Fisher Scientific) was employed to detect the concentration of NH_4_^+^ and the anions NO_3_^–^ and NO_2_^–^. The isotopic nitrogen test was measured via the No-deuterium proton nuclear magnetic resonance (No-D NMR) spectroscopy measurement using the JEOL ECZ500R/S1 instrument. In situ Raman spectrometry (IHR-550; HORIBA) was used to measure the bonding vibration modes and the bonding condition of MoS_2_ to the N_2_ molecule. In situ diffuse reflectance infrared Fourier-transform spectroscopy (DRIFTS, Vertex80v; Bruker) was used to detect the byproduct of the piezocatalytic N_2_ reduction reaction and the absorption behavior of V2s-MoS_2_. Mo K-edge XANES and EXAFS spectra for V2s-MoS_2_ were obtained at the TPS44A beamline of the National Synchrotron Radiation Research Center (NSRRC).

### Computational Details of Density Functional Theory (DFT)

In this study, we implement density functional theory (DFT) based *ab initio* calculations via Vienna *ab initio* simulation package (VASP).^54^ For the exchange-correlation functional, the general gradient approximation (GGA) of the Perdew–Burke–Ernzerhof scheme (PBE) was utilized.^55^ The ion-electron interaction was described by the projected-augmented-wave method (PAW) with an energy cutoff of 600 eV.^56^ To account for the weak correlation, the rev-vdW-DF2 functional by Hamada was employed for van der Waals (vdW) interactions.^57^ The *k*-point mesh of 12 × 12 × 1 and 2 × 2 × 1 is used to sample the Brillouin zone of (1 × 1) and (6 × 6) MoS_2_, respectively. The vacuum layer is larger than 20 Å. The tetrahedron method was used for the density of states calculations. The optimized lattice constant of MoS_2_ is 3.17 Å. To investigate the interaction between MoS_2_ and N_2_, we evaluate the adsorption energy (*E*_ads_) under different percentages of strain, including −10, −5, 0, +5, and +10%. The negative sign denotes the compressive strain, and the positive sign denotes the tensile strain. The *E*_ads_ was evaluated with the equation:

$$E_{\mathrm{ads}}=E_{\mathrm{system}}-E_{\mathrm{substrate}}-\mu_{\mathrm{adsorbate}}$$

where the *E*_system_, *E*_substrate_, and μ_*adsorbate*_ correspond to the total energy of the system, energy of the substrate material, and the chemical potential of the adsorbate, respectively. Furthermore, with a view to evaluating the activity under different strain conditions, the Gibbs free energy change (Δ*G*) for NRR is calculated using the equation:

$$\Delta G=E_{\mathrm{ads}}+\Delta E_{\mathrm{ZPE}}-T\Delta S$$

where the Δ*E*_ZPE_ and *T*Δ*S* are the difference in zero-point correction and the entropy contribution of adsorbates at room temperature of 298.15K. The Δ*E*_ZPE_ was evaluated within the finite difference approach implemented in VASP. With a view to analyzing the charge transfer process throughout the reaction, the electron density difference (Δρ) and Bader charge analysis developed by Henkelman were utilized.^58^ The electronic density difference can be calculated by

$$\Delta \rho =\rho_{\mathrm{system}}-\rho_{\mathrm{substrate}}-\rho_{\mathrm{adsorbate}}$$

where the ρ_system_, ρ_substrate_, and ρ_adsorbate_ represent the charge densities of the system, substrate, and adsorbate, respectively.

## Supplementary Material





## Notes

SEM images, HRTEM images, DFT calculations, and BET results (PDF)
MoS_2_ side-on animation (AVI)
