# Supplementary material for: Direct and Sustainable Ammonia Synthesis from Air and Water with Sulfur-Deficient MoS2 Piezocatalysts
Source: ACS Nano. 2025 Oct 6;19(41):36602–13. doi: 10.1021/acsnano.5c11903 (PMC12548343; doi:10.1021/acsnano.5c11903)
Supplement: Supplementary file 1 [file nn5c11903_si_001.pdf]

Supporting Information

Direct and Sustainable Ammonia Synthesis from Air and Water with Sulfur-Deficient MoS_2 Piezocatalysts

Yu-Ching Chen[1, 2], Yin-Song Liao[4,5], Po-Han Chen[1], Jyh-Pin Chou[5, 6], Cheng-Kuo Tsai[7], Yi-Dong Lin[8], Yan-Gu Lin[9], Yu-Ren Peng[1,2] and Jyh Ming Wu[*, 1,3]

[1]Department of Materials Science and Engineering, National Tsing Hua University, 101, Section 2 Kuang Fu Road, Hsinchu 300, Taiwan.

[2]Ph.D. Program in Prospective Functional Materials Industry, National Tsing Hua University, 101, Section 2 Kuang Fu Road, Hsinchu 300, Taiwan.

[3] High Entropy Materials Center, National Tsing Hua University, 101, Section 2 Kuang Fu Road, Hsinchu 300, Taiwan.

[4] Tsing Hua Interdisciplinary Program, National Tsing Hua University, 101, Section 2 Kuang Fu Road, Hsinchu 300, Taiwan.

[5]Graduate School of Advanced Technology, National Taiwan University, Taipei 106319, Taiwan.

[6]Department of Physics, National Changhua University of Education, No. 1, Jin-De Road, Changhua 500, Taiwan.

[7]Emergency Response Information Center, National Yunlin University of Science and Technology, Douliu City, Yunlin County, 64002, Taiwan

[8]Institute of Pioneer Semiconductor Innovation, National Yang Ming Chiao Tung University, Hsinchu, 300 Taiwan.

[9]National Synchrotron Radiation Research Center, 101 Hsin-Ann Road, Hsinchu Science Park, Hsinchu, 300 Taiwan.

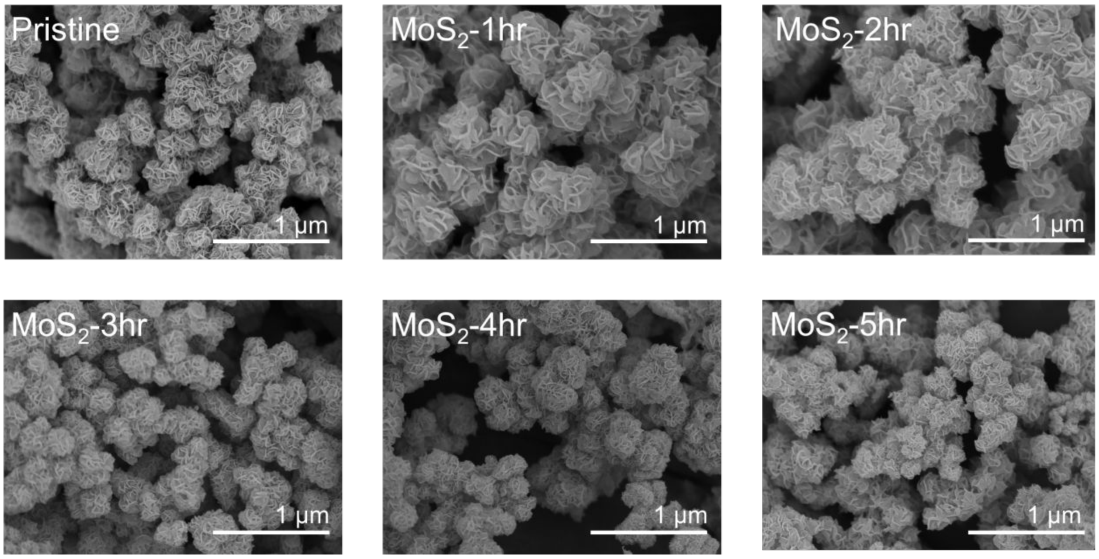

Figure S1. SEM images of MoS_2 NFs under different Ar/H_2 annealing times.

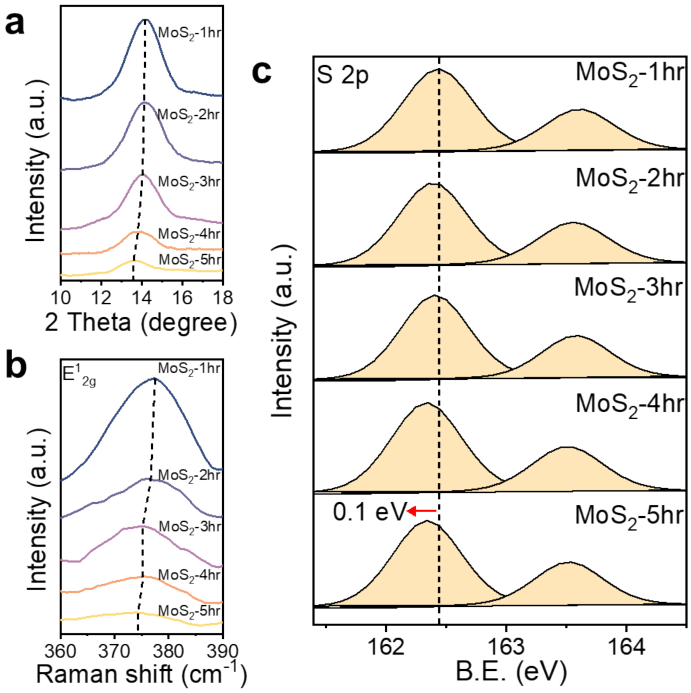

Figure S2. (a) Localized magnification of MoS_2 (002) plane, illustrating a shift to a higher angle as annealing time varies. (b) Localized magnification of the Raman spectra showing a shift in the E^1_{2g} peak. (c) S 2p peak in XPS, shifting to lower binding energy due to sulfur vacancies.

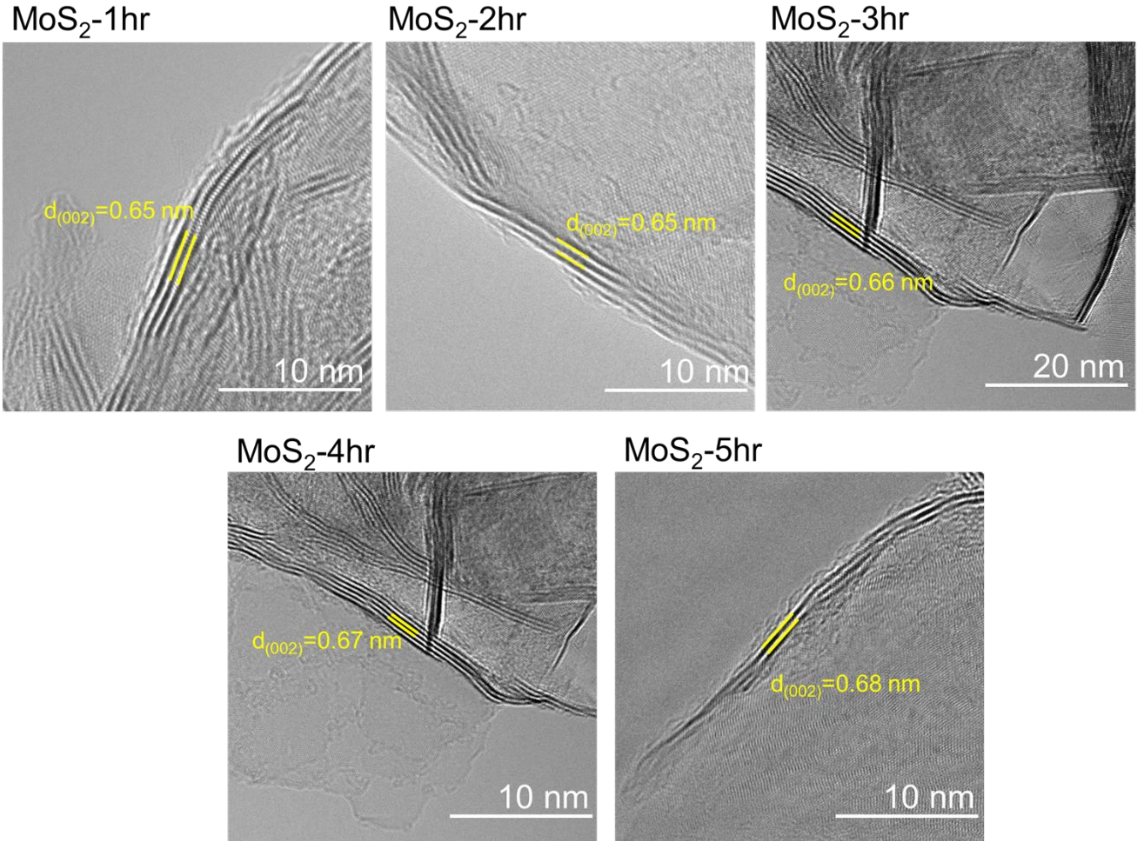

Figure S3. High resolution TEM of different MoS_2 samples.

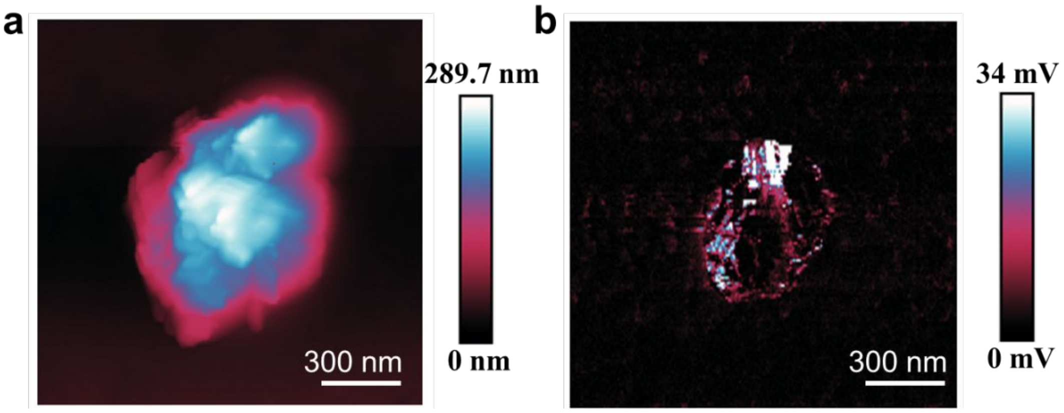

Figure S4. (a) 2D contour map of the V2s MoS_2-3hr NFs. (b) PFM results corresponding to the V2s MoS_2-3hr NFs.

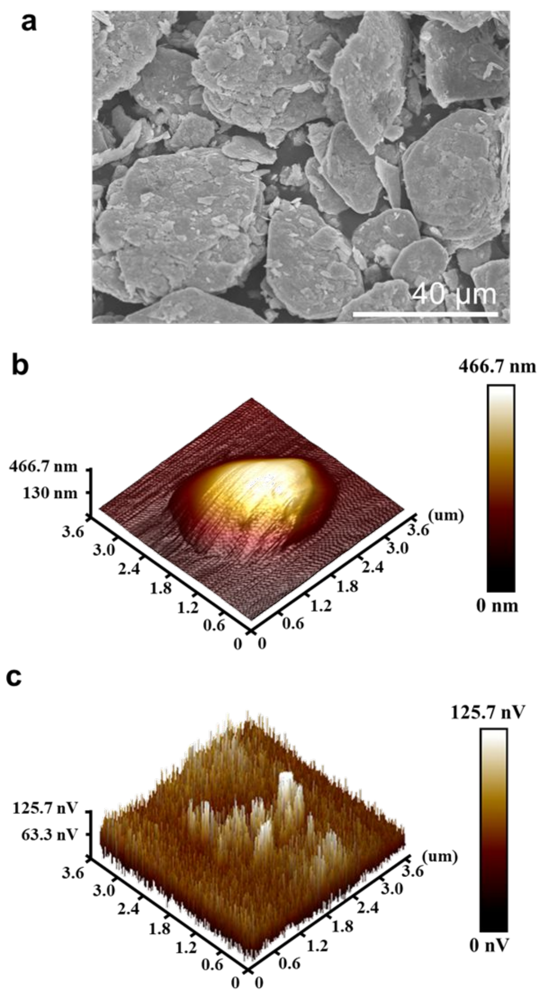

Figure S5. (a) SEM image of commercial MoS_2 (b) 3D topographic of commercial MoS_2. (c) PFM result of commercial MoS_2.

Supporting Information of S1

We estimated an effective d_{33} values derived from PFM amplitude data using Equation (1) as follows:

$$A = d_{33} \times V_{AC} \tag{1}$$

where A is the PFM amplitude and V_{AC} is the AC driving voltage. The calculated effective d_{33} value is 33.5 pm/V. We emphasize that the effective d_{33} value, measured under strong-indentation PFM. They reflect the vertical piezoelectric response but do not correspond to the intrinsic d_{33} tensor component, which is theoretically zero in ideal 2H-MoS_2 due to its symmetry. However, an effective d_{33}—not the intrinsic out-of-plane

coefficient—due to the nature of our sample geometry. Owing to the D_{3h} crystal symmetry of monolayer and odd-layer 2H-MoS_2, intrinsic piezoelectricity is restricted to the in-plane directions (x, y), and the true d_{33} component is theoretically zero in ideal monolayers. Yet, in few-layer nanostructures with out-of-plane curvature, inclined stacking, and edge terminations, in-plane components such as d_{11} and d_{12} can be projected into the vertical (z-axis) direction during PFM measurements. Therefore, the observed vertical piezoresponse, while reported as an effective d_{33}, is partially contributed by in-plane piezoelectricity aligned with local strain directions. This complex projection is particularly relevant in our defect-rich nanoflower structures, which do not have perfect basal plane alignment.

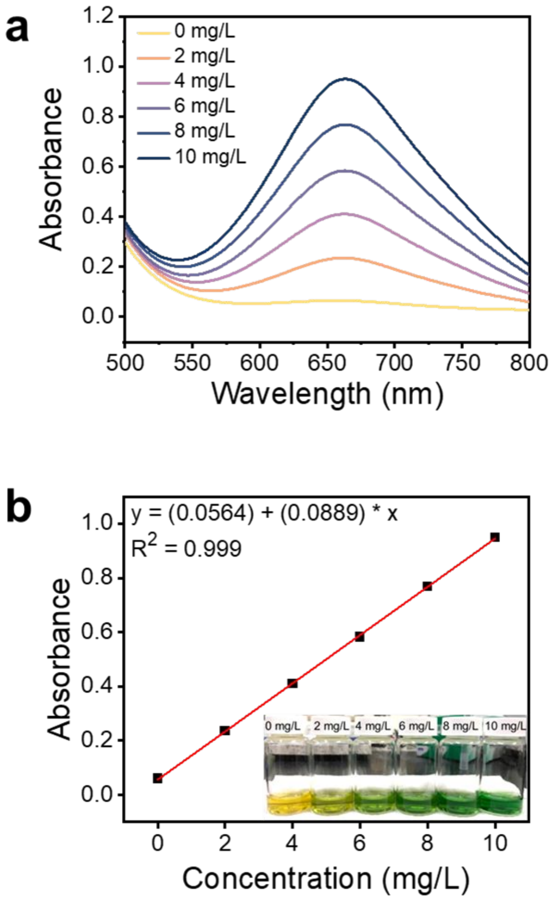

Figure S6. Indophenol blue method (a) UV-vis absorption spectra and (b) calibration curve.

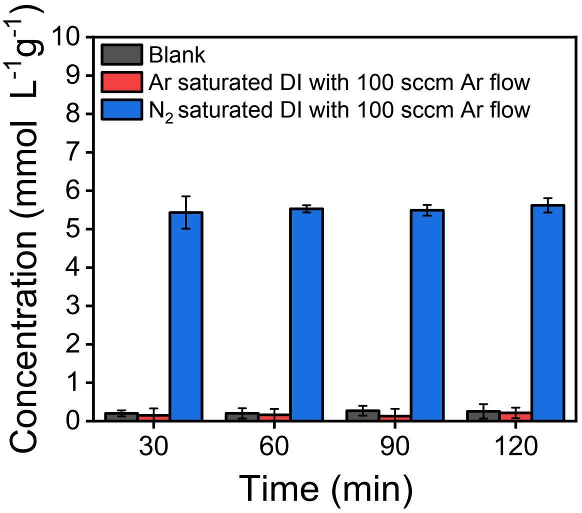

Figure S7. The NH_3 yield rate of MoS_2-3hr under different pre-saturation conditions with DI water and a 100 sccm Ar flow and blank test (without catalysts).

Supporting Information of S2

Figure S8a show the optical image of 8-hour long stability test using IPB method and Figures S8b and S8c show the UV-vis spectra from an 8-hour long-term stability test of MoS_2-3hr, using air and pure N_2 as feed gases, respectively. The piezocatalytic NH_3 production results for both air and N_2 show an increasing trend, with the highest absorbance values reaching 0.45 and 0.64, respectively, at the eighth hour. The performance with N_2 as the feeding gas is 1.4 times higher than that with air.

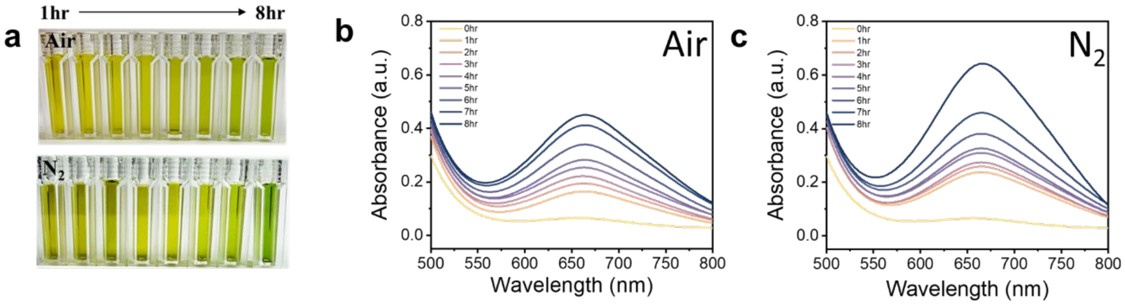

Figure S8. (a) Optical images showing the results of an 8-hour long-term stability test using IPB method. UV-vis spectra of 8 hours piezocatalytic NRR with (b) air and (c) N_2 as feeding gas.

Supporting Information of S3

As shown in Figures S9a and S9b, the SEM and STEM images of the MoS_2-3hr catalyst before the piezocatalytic reaction display a well-defined nanoflower morphology with a clearly defective surface structure. After the piezocatalytic reaction (Figures S9c and S9d), the overall morphology remains intact without noticeable deformation or collapse, indicating excellent structural stability. In addition, the EPR spectra (Figure S9e) demonstrate that the signal intensity associated with sulfur vacancies remains nearly unchanged before and after the reaction, confirming the persistence of defect sites. Raman spectra (Figure S9f) further verify that the MoS_2 nanoflowers retain their characteristic 2H phase throughout the catalytic process. Meanwhile, XPS spectra of the Mo 3d and S 2p regions (Figures S9g and S9h) exhibit no significant shifts in binding energy or variation in peak intensity, indicating that the sulfur vacancies are chemically stable and not passivated or restored during the reaction. Collectively, these comprehensive characterizations confirm that the engineered sulfur vacancies in MoS_2-3hr remain both structurally and chemically stable under catalytic conditions, continuing to function as active sites for N_2 activation.

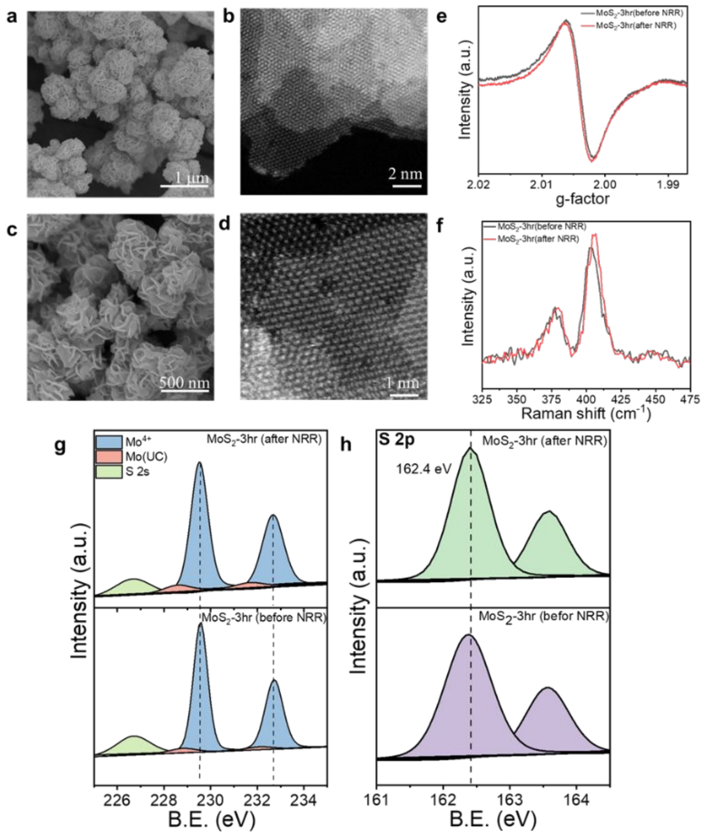

Figure S9. (a) and (b) SEM and STEM images of MoS_2-3hr before the piezocatalytic reaction, showing nanoflower morphology and clear lattice fringes. (c) and (d) corresponding SEM and STEM images after the piezocatalytic reaction. (e) EPR spectra before and after piezocatalysis, showing nearly identical sulfur vacancy signal intensities. (f) Raman spectra confirming the retention of the 2H phase of MoS_2 before and after the reaction. (g) XPS spectra of Mo 3d and (h) S 2p regions.

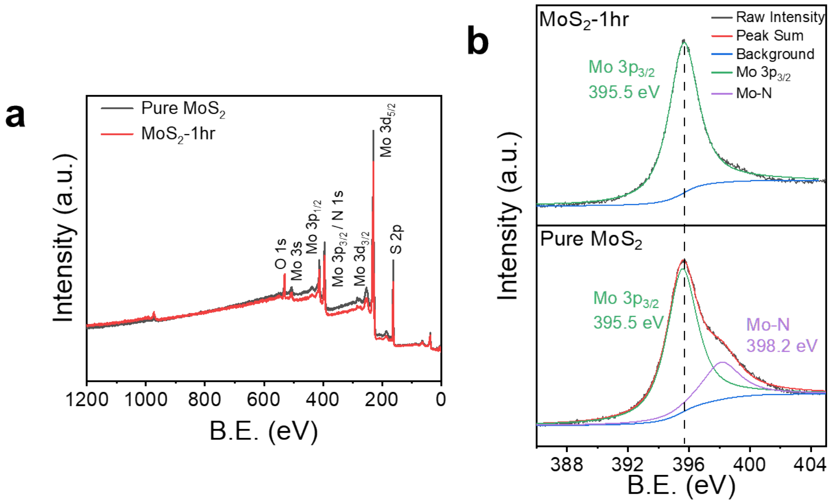

Figure S10. (a) XPS survey spectra of pure MoS_2 and Ar/H_2-treated MoS_2-1hr. (b) High-resolution N 1s spectra of pure MoS_2 and Ar/H_2-treated MoS_2-1hr.

Supporting Information of S4

Given that a nitrogen-containing precursor, thiourea, was used during the material synthesis process, we conducted a long-term soaking test to evaluate the potential presence of residual ammonia in the catalyst. The experimental setup was scaled up from the original nitrogen reduction reaction conditions, increasing the catalyst amount from 10 mg (in 10 mL of N_2-saturated deionized water) to 100 mg of MoS_2-1hr in 50 mL. To eliminate the possibility of light-induced photocatalytic effects, the reactor was shielded with an alumina cover. Additionally, ultrasonic vibration used in the previous setup was replaced with continuous magnetic stirring to facilitate the release of any residual ammonia. Under these conditions, if trace amounts of ammonia remained within the MoS_2, they would be expected to gradually diffuse into the N_2-saturated solution over the 7-day stirring period, potentially leading to detectable signals. To monitor any product evolution, 1 mL of solution was sampled daily and analyzed using ^{1}H-NMR to detect the presence of ammonia. As shown in Figure S11, no signals corresponding to NH_4^+ were observed from day 1 to day 7; identification

was confirmed by comparison with a standard solution of NH_4Cl. These results indicate that the thermal treatment applied to generate sulfur vacancies was effective in removing nitrogen-containing residues originating from the thiourea precursor used during catalyst synthesis.

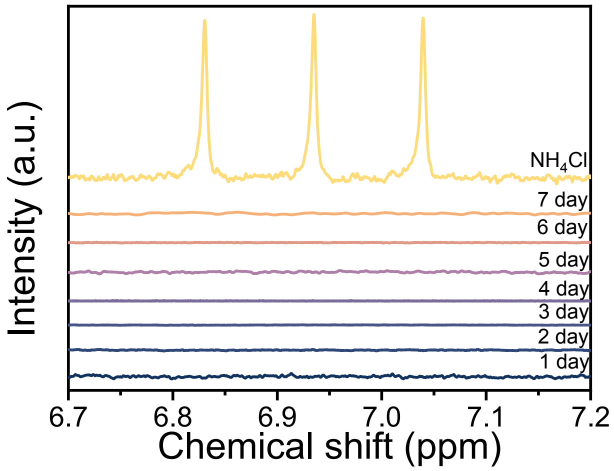

Figure S11. ^{1}H-NMR spectra of daily samples from a 7-day soaking test of MoS_2-1hr in N_2-saturated DI water and standard NH_4^+ peak prepared by NH_4Cl. No NH_4^+ signals were detected, indicating effective removal of nitrogen residues after thermal treatment.

Supporting Information of S5

Figure S12a presents the ^{1}H-NMR spectrum using NH_4Cl as the calibration standard. Figure S12b displays the calculated calibration results, with a regression coefficient of 0.999, indicating excellent linearity.

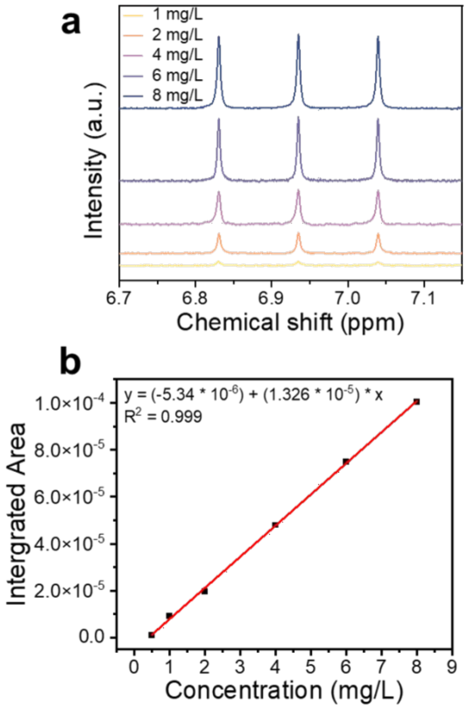

Figure S12. (a) ^{1}H-NMR spectra of $^{14}NH_4^+$ in various concentrations and (b) calibration curve.

Supporting Information of S6

Figure S13a shows the ion chromatography profiles measured using different concentration commercial standards, with the target ammonium ion primarily appearing at the fifth minute. Figure S13b presents the calculated regression line with a regression coefficient of 0.998. Figure S13c presents the IC results for the optimized MoS_2-3hr piezocatalyst after 2 hours of piezocatalysis, where two distinct characteristic peaks corresponding to Na^+ and NH_4^+ are observed. The Na^+ peak is likely attributed to residual sodium originating from the sodium molybdate precursor used during the catalyst synthesis. In addition to the tested sample, two control spectra are also included in Figure S13c for comparison: one for pure DI water and another for DI water saturated with N_2 gas. Neither control sample exhibits detectable NH_4^+ signals, confirming that the observed NH_4^+ peak in the MoS_2-3hr sample arises from the catalytic conversion of N_2 and is not the result of background contamination.

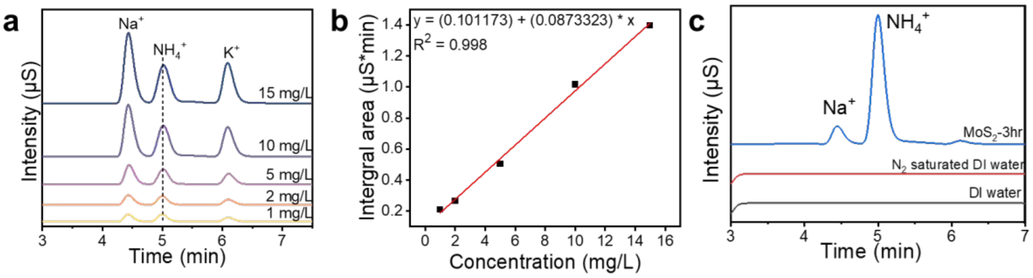

Figure S13. (a) The spectra of ion chromatography for the standard solution of $^{14}NH_4^+$ with various concentrations and (b) calibration curve. (c) The spectra illustrate the solution of pure DI water, DI water saturated with N_2, and post-piezocatalysis NRR of MoS_2-3hr

Supporting Information of S7

Hydrazine, as one of the potential intermediates, was monitored using the Watt-Chrisp method and UV-vis spectroscopy to determine its presence. Figures S14a and S14b display the UV spectra and the regression line obtained from standard samples, respectively, with the inset in Figure S14b showing the color changes at different concentrations. Figure S14c demonstrates that after two hours of ultrasonic-driven piezocatalytic reactions, no characteristic peak at 460 nm was observed for any of the defective MoS_2 samples, indicating no hydrazine formation and thus excellent selectivity.

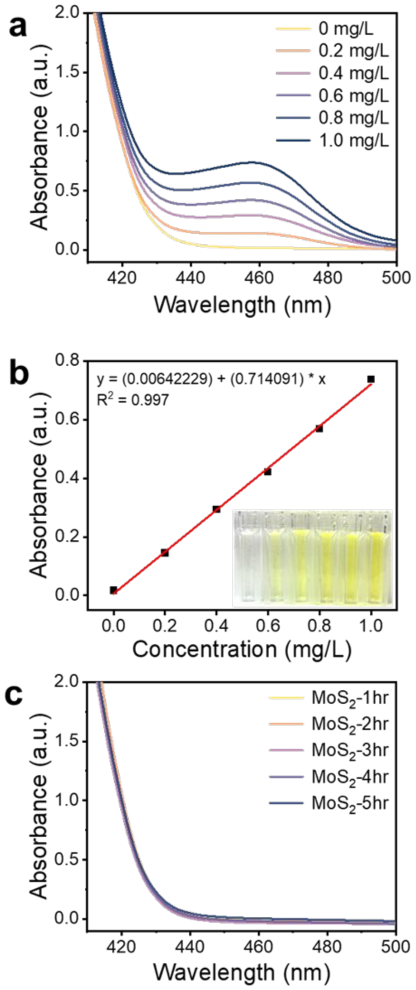

Figure S14. The Watt-Chrisp method (a) UV-vis absorption spectra and (b) calibration curve. (c) Hydrazine detection results of different MoS_2 samples after NRR reaction.

Supporting Information of S8

Figures S15a-S15d show the calibration curves for $NaNO_2$ (S15a and S15b) and $NaNO_3$ (S15c and S15d), detected via anion IC. Figure S15e indicates that the use of an nitrogen oxide (NO_x) trap gas purification system effectively removes potential NO_x contaminants from the water source or feed gas, thereby eliminating interference and purifying the gas.

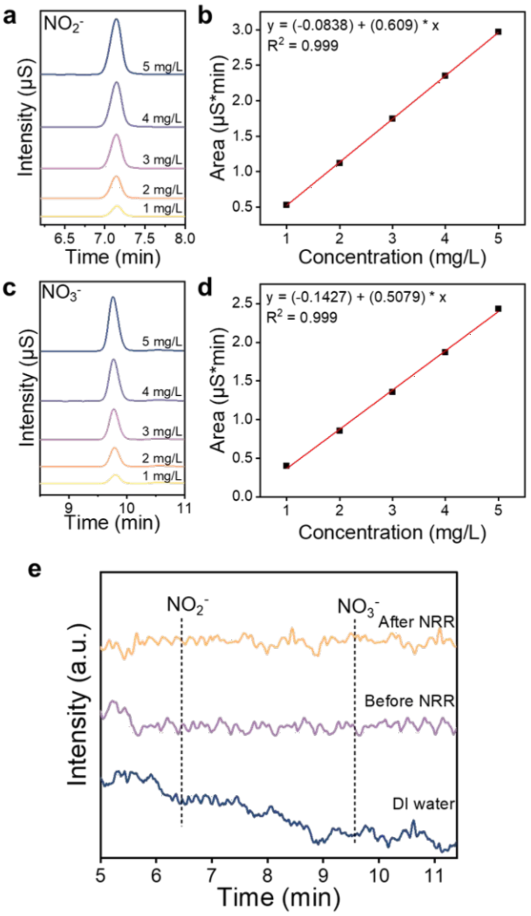

Figure S15. IC spectra of (a) different concentrations of $NaNO_2$, (b) calibration curve of NO_2^-, (c) different concentrations of $NaNO_3$, and (d) calibration curve of NO_3^-. (e) The spectra illustrate the solution of pure DI water, DI water saturated with N_2 prepared before NRR, and post-piezocatalysis NRR.

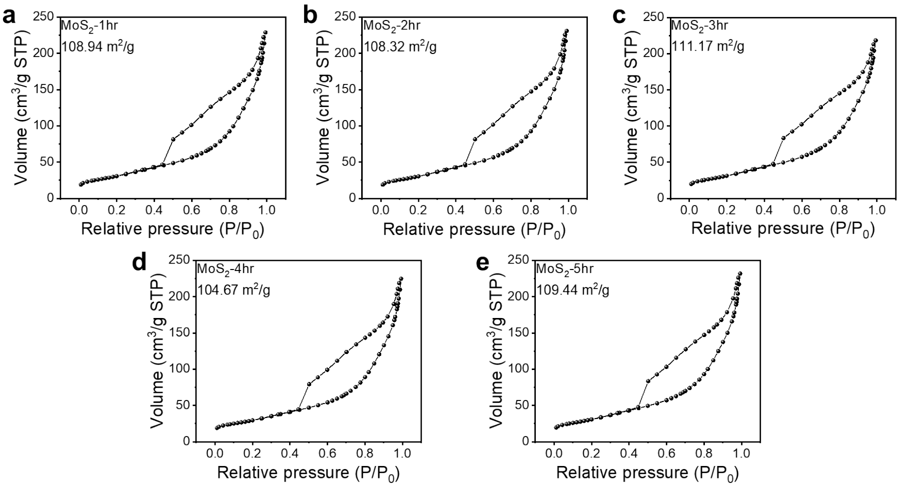

Figure S16. BET results of (a) MoS_2-1hr, (b) MoS_2-2hr, (c) MoS_2-3hr, (d) MoS_2-4hr,

(e) MoS_2-5hr.

Supporting Information of S9

In Figure S17, we analyzed N_2 adsorption in both side-on and end-on configurations under strains of –10%, –5%, 0%, +5%, and +10%. In the absence of strain, all three scenarios exhibit positive adsorption energies, suggesting that N_2 molecules are unlikely to adsorb on the V2s-MoS_2 surface. Subsequently, tensile and compressive strains are applied to all models to simulate the environmental stress experienced by the V2s-MoS_2 NFs. The initially approached N_2 molecule adopts an end-on configuration toward the V2s-MoS_2, yet ultimately transitions to a side-on configuration after structure optimization (Video I, Supporting Information).

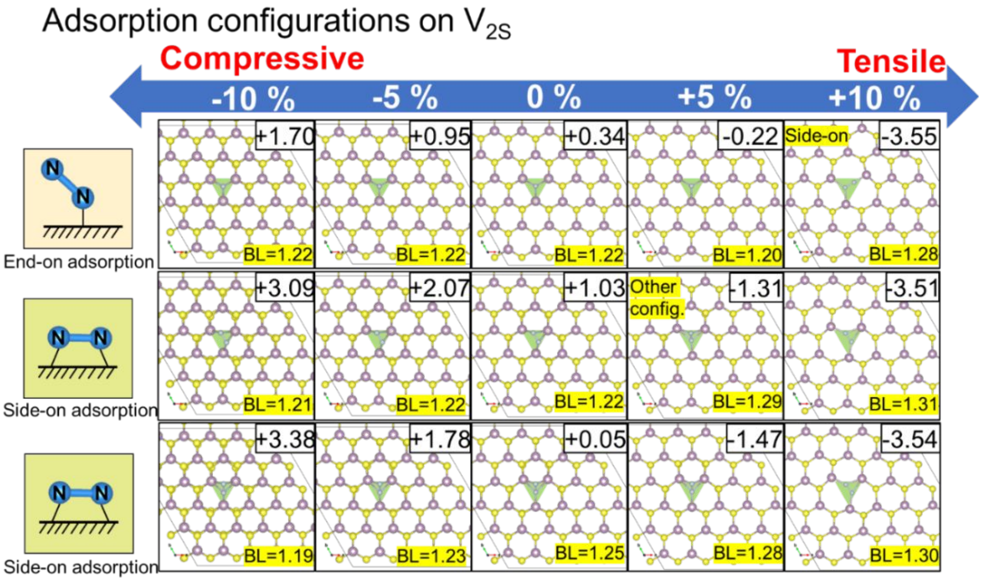

Figure S17. Changes in the adsorption energy of N_2 molecule for end-on and side-on configurations under various strain conditions.

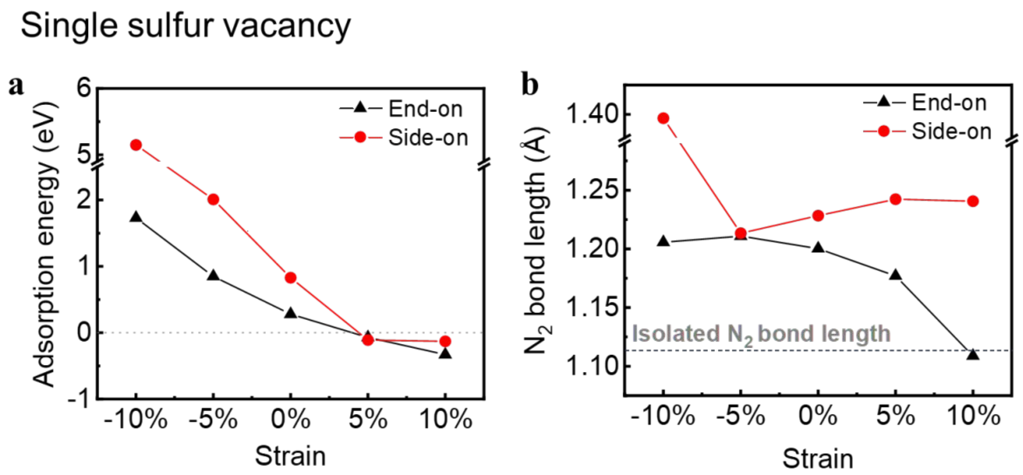

Figure S18. (a) Effect of compressive and tensile strain on the adsorption energy of single sulfur vacancy MoS_2. (b) Relationship between N_2 bond length and adsorption on single sulfur vacancy MoS_2 under compressive and tensile strain.

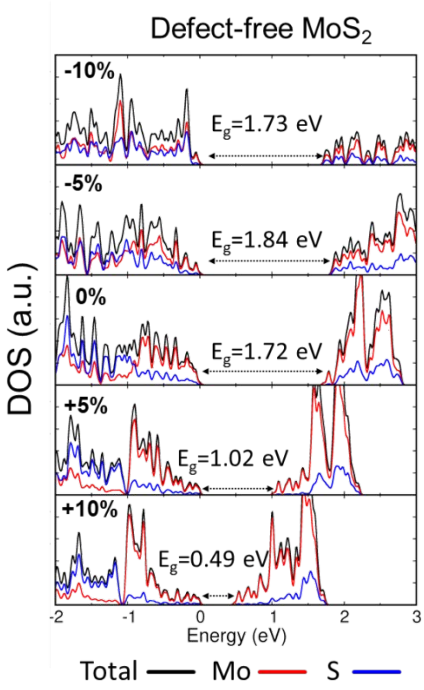

Figure S19. Total DOS of defect-free MoS_2 under different percentages of strain.

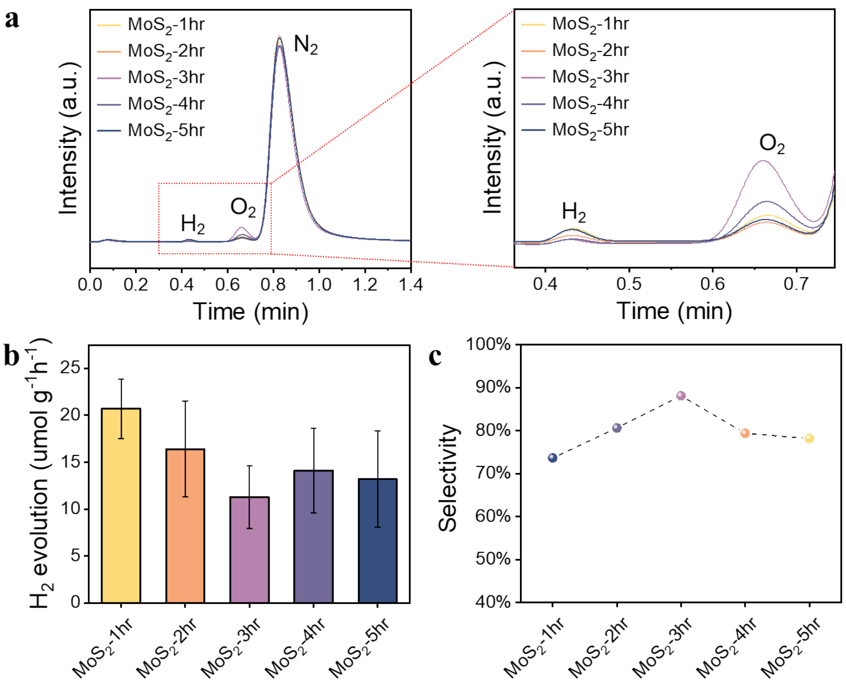

Figure S20. (a) GC spectra of various V2s-MoS_2 samples following a 2-hour piezocatalysis reaction. And the Zoomed-in region highlighted by the red dashed box. (b) The H_2 yield rate for the different annealing times of MoS_2 NFs. (c) Calculated selectivity of MoS_2 NFs.

Supporting Information of S10

In the piezocatalytic system developed in this study, V2s-defective MoS_2 nanoflowers generate an internal electric field under mechanical strain (e.g., ultrasonic vibration) via the piezoelectric effect, as shown in Equation (2):

$$\text{V2s MoS}_2 + \text{mechanical strains} \xrightarrow{Electrical\ field} e^- + h^+ \qquad (2)$$

This internal field effectively promotes the separation of electron–hole pairs, thereby initiating two primary reaction pathways: nitrogen reduction (NRR) and water oxidation. The generated electrons (e^-) participate in the stepwise hydrogenation of adsorbed nitrogen molecules, ultimately producing ammonia. The presence of dual sulfur vacancies plays a crucial role in enhancing the adsorption and activation of N_2, enabling efficient NRR under ambient conditions.

Simultaneously, the piezo-generated holes (h^+) oxidize water molecules to produce highly reactive hydroxyl radicals (•OH), as described in Equation (3):

$$H_2O + h^+ \leftrightarrow \bullet OH + H^+ \quad (3)$$

These •OH radicals further recombine to form water and molecular oxygen, as shown in Equation (4):

$$2\bullet OH \leftrightarrow \frac{1}{2}O_2 + H_2O \quad (4)$$

Through this mechanism, the piezocatalytic system enables simultaneous nitrogen reduction and water oxidation without requiring external bias or sacrificial agents. The main products are NH_3 from the reduction pathway and O_2 from the oxidation pathway. The formation of •OH radicals has been confirmed by electron paramagnetic resonance spectroscopy in this study, while the evolution of oxygen and other oxidation byproducts was validated by gas chromatography, as shown in Figure S20. These results provide strong evidence for the radical-mediated oxidation mechanism.

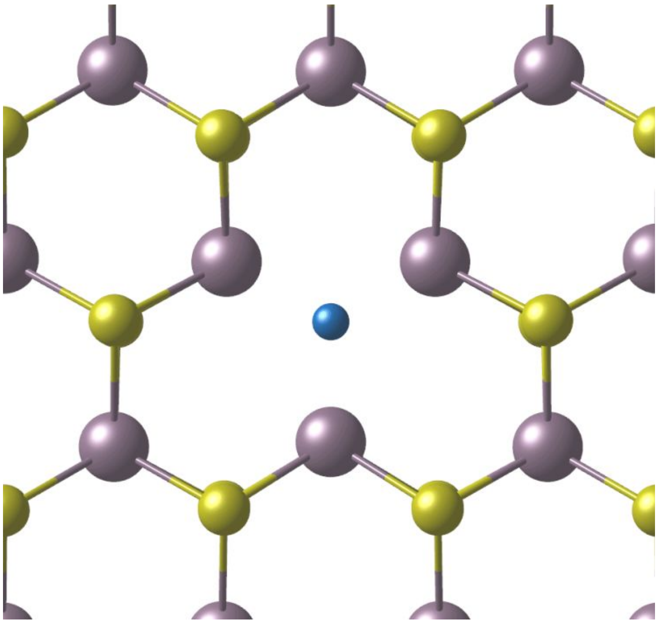

Video I. The optimization of initially end-on N_2 adsorbed on V2s-MoS_2 with +10% tensile strain becomes side-on configuration finally.

Table S1. Comparative summary of catalyst dosage, reaction conditions, and absolute ammonia production rates in reported NRR systems.

**Catalyst**	**Mechanism**	**NH_3 yield rate (μmol $L^{-1}g^{-1}h^{-1}$)**	**Catalyst dosage (g)**	**Solution volume**	**Ref.**
V2s-MoS_2	Piezocatalysis	8374.8 (0.84 μmol h^{-1})	0.01	10 mL DI water	This work
ZnO	Piezocatalysis	21.44 (0.15 μmol h^{-1})	0.07	70 mL DI water	1
$KTa_{0.5}Nb_{0.5}O_3$	Piezocatalysis	13.2 (0.066 μmol h^{-1})	0.1	5 mL MeOH + 95 mL DI water	2
2.5% Ag/Bi_5O_7I	Piezocatalysis	65.4 (0.065 μmol h^{-1})	0.05	200 mL Na_2SO_4 (0.5 mol/L)	3
CuS/ZnO	Piezocatalysis	77.5	0.005	Unknow	4
$CuS/KTa_{0.75}Nb_{0.25}O_3$	Piezocatalysis	36.2 (0.181 μmol h^{-1})	0.1	5 mL MeOH + 95 mL DI water	5
$Bi_2S_3/KTa_{0.75}Nb_{0.25}O_3$	Piezocatalysis	14.9 (0.075 μmol h^{-1})	0.05	5 mL MeOH + 95 mL DI water	6

$BaTiO_3$-O_v	Piezocatalysis	498.33 (0.59 μmol h^{-1})	0.02	60 mL (0.1 M Na_2S with 0.1 M Na_2SO_3)	7
TiO_2/Au/BiOI	Photocatalysis	543.53 (0.54 μmol h^{-1})	0.01	100 mL DI water	8
7.5% Bi-Bi_2O_3/$CdWO_4$	Photocatalysis	434.9 (4.35 μmol h^{-1})	0.1	5 mL MeOH + 95 mL DI water	9
$BiPO_4$/$Bi_4O_5Br_2$	Photocatalysis	370 (3.7 μmol h^{-1})	0.1	5 mL MeOH + 95 mL DI water	10
7.5% $NaNbO_3$/$Bi_2O_2CO_3$	Photocatalysis	453.1 (9.06 μmol h^{-1})	0.1	10 mL MeOH + 190 mL DI water	11
$NiP_{x\text{-}3}$DOM H_xWO_{3-y}	Photocatalysis	680 (0.85 μmol h^{-1})	0.025	2 mL MeOH + 48 mL DI water	12
ZnS/GO	Photocatalysis	151.7 (0.76 μmol h^{-1})	0.05	100 mL DI water	13
Au/TiO_2	Photocatalysis	78.6 (7.86 μmol h^{-1})	0.1	8 mL MeOH + 72 mL DI water	14

MoS_2/C-ZnO	Photocatalysis	245.7 (4.91 μmol h^{-1})	0.1	10 mL MeOH + 190 mL DI water	15
{[Zn(L)(N_2)$_{0.5}$(TCNQ–TCNQ)$_{0.5}$]·(TCNQ)$_{0.5}$}$_n$	Photocatalysis	2333.4 (2.8 μmol h^{-1})	0.02	60 mL DI water	16
MoO_{3-x}	Photocatalysis	1393.4 (4.18 μmol h^{-1})	0.02	150 mL DI water	17
TiO_2-O_v	Photocatalysis	3945 (1.58 μmol h^{-1})	0.02	20 mL DI water	18

Table S2. Cross-sectional analysis of the thickness of MoS_2-3hr.

Sample	Vertical Distance (nm)
Line 1	1.864
Line 2	1.932
Line 3	2.041
Line 4	1.977
Line 5	1.985

Avg.	1.959

References

1. Peng, F.; Lin, J.; Li, H.; Liu, Z.; Su, Q.; Wu, Z.; Xiao, Y.; Yu, H.; Zhang, M.; Wu, C.; Wang, W.; Lu, C., Design of piezoelectric ZnO based catalysts for ammonia production from N2 and H2O under ultrasound sonication. *Nano Energy* **2022,** *95*, 107020.
2. Chen, L.; Wang, J.; Li, X.; Zhang, J.; Zhao, C.; Hu, X.; Lin, H.; Zhao, L.; Wu, Y.; He, Y., Facile preparation of Ag2S/KTa0.5Nb0.5O3 heterojunction for enhanced performance in catalytic nitrogen fixation via photocatalysis and piezo-photocatalysis. *Green Energy Environ.* **2023,** *8* (6), 1630-1643.
3. Chen, L.; Zhang, W.; Wang, J.; Li, X.; Li, Y.; Hu, X.; Zhao, L.; Wu, Y.; He, Y., High piezo/photocatalytic efficiency of Ag/Bi5O7I nanocomposite using mechanical and solar energy for N2 fixation and methyl orange degradation. *Green Energy Environ.* **2023,** *8* (1), 283-295.
4. Ning, X.; Jia, D.; Li, S.; Khan, M. F.; Hao, A., Construction of CuS/ZnO Z-scheme heterojunction as highly efficient piezocatalyst for degradation of organic pollutant and promoting N2 fixation properties. *Ceram. Int.* **2023,** *49* (13), 21658-21666.
5. Dai, X.; Chen, L.; Li, Z.; Li, X.; Wang, J.; Hu, X.; Zhao, L.; Jia, Y.; Sun, S.-X.; Wu, Y.; He, Y., CuS/KTa0.75Nb0.25O3 nanocomposite utilizing solar and mechanical energy for catalytic N2 fixation. *J. Colloid Interface Sci.* **2021,** *603*, 220-232.
6. Chen, L.; Dai, X.; Li, X.; Wang, J.; Chen, H.; Hu, X.; Lin, H.; He, Y.; Wu, Y.; Fan, M., A novel Bi2S3/KTa0.75Nb0.25O3 nanocomposite with high efficiency for photocatalytic and piezocatalytic N2 fixation. *J. Mater. Chem. A* **2021,** *9* (22), 13344-13354.
7. Yuan, J.; Feng, W.; Zhang, Y.; Xiao, J.; Zhang, X.; Wu, Y.; Ni, W.; Huang, H.; Dai, W., Unraveling Synergistic Effect of Defects and Piezoelectric Field in Breakthrough Piezo-Photocatalytic N2 Reduction. *Adv. Mater.* **2024,** *36* (5), 2303845.
8. Yu, X.; Qiu, H.; Wang, Z.; Wang, B.; Meng, Q.; Sun, S.; Tang, Y.; Zhao, K., Constructing the Z-scheme TiO2/Au/BiOI nanocomposite for enhanced photocatalytic nitrogen fixation. *Appl. Surf. Sci.* **2021,** *556*, 149785.
9. Mao, J.; Liu, H.; Cui, X.; Zhang, Y.; Meng, X.; Zheng, Y.; Chen, M.; Pan, Y.; Zhao, Z.; Hou, G.; Hu, J.; Li, Y.; Xu, G.; Huang, R.; Yu, L.; Deng, D., Direct conversion of methane with O2 at room temperature over edge-rich MoS2. *Nat. Catal.* **2023,** *6* (11), 1052-1061.

10. Zhao, C.; Li, X.; Yue, L.; Ren, X.; Yuan, S.; Zeng, Z.; He, Y., Fabrication of novel BiPO4/Bi4O5Br2 heterojunctions for improving photoactivity in N2 fixation and dye degradation. *Mater. Res. Bull.* **2023,** *167*, 112377.
11. Yue, L.; Zhang, J.; Zeng, Z.; Zhao, C.; Hu, X.; Zhao, L.; Zhao, B.; He, Y., In Situ Fabrication of an S-Scheme NaNbO3/Bi2O2CO3 Heterojunction for Enhanced Performance in Photocatalytic Nitrogen Fixation. *Langmuir* **2023,** *39* (37), 13267-13278.
12. Ren, X.; Xia, M.; Chong, B.; Yan, X.; Wells, N.; Yang, G., Uniform NiPx nanospheres loaded onto defective HxWO3-y with three-dimensionally ordered macroporous structure for photocatalytic nitrogen reduction. *Appl. Catal., B* **2021,** *297*, 120468.
13. Wang, S.-X.; Maimaiti, H.; Xu, B.; Awati, A.; Zhou, G.-B.; Cui, Y.-d., Synthesis and visible-light photocatalytic N2/H2O to ammonia of ZnS nanoparticles supported by petroleum pitch-based graphene oxide. *Appl. Surf. Sci.* **2019,** *493*, 514-524.
14. Yang, J.; Guo, Y.; Jiang, R.; Qin, F.; Zhang, H.; Lu, W.; Wang, J.; Yu, J. C., High-Efficiency "Working-in-Tandem" Nitrogen Photofixation Achieved by Assembling Plasmonic Gold Nanocrystals on Ultrathin Titania Nanosheets. *J. Am. Chem. Soc.* **2018,** *140* (27), 8497-8508.
15. Xing, P.; Chen, P.; Chen, Z.; Hu, X.; Lin, H.; Wu, Y.; Zhao, L.; He, Y., Novel Ternary MoS2/C-ZnO Composite with Efficient Performance in Photocatalytic NH3 Synthesis under Simulated Sunlight. *ACS Sustainable Chem. Eng.* **2018,** *6* (11), 14866-14879.
16. Xiong, Y.; Li, B.; Gu, Y.; Yan, T.; Ni, Z.; Li, S.; Zuo, J.-L.; Ma, J.; Jin, Z., Photocatalytic nitrogen fixation under an ambient atmosphere using a porous coordination polymer with bridging dinitrogen anions. *Nat. Chem.* **2023,** *15* (2), 286-293.
17. Liu, X.; Luo, Y.; Ling, C.; Shi, Y.; Zhan, G.; Li, H.; Gu, H.; Wei, K.; Guo, F.; Ai, Z.; Zhang, L., Rare earth La single atoms supported MoO3-x for efficient photocatalytic nitrogen fixation. *Appl. Catal., B* **2022,** *301*, 120766.
18. Zhao, Y.; Zhao, Y.; Shi, R.; Wang, B.; Waterhouse, G. I. N.; Wu, L.-Z.; Tung, C.-H.; Zhang, T., Tuning Oxygen Vacancies in Ultrathin TiO2 Nanosheets to Boost Photocatalytic Nitrogen Fixation up to 700 nm. *Adv. Mater.* **2019,** *31* (16), 1806482.
